# Disentanglement of single-cell data with biolord

**DOI:** 10.1038/s41587-023-02079-x

**Published:** 2024-01-15

**Authors:** Zoe Piran, Niv Cohen, Yedid Hoshen, Mor Nitzan

**Affiliations:** 1 School of Computer Science and Engineering, The Hebrew University, Jerusalem, Israel; 2 Racah Institute of Physics, The Hebrew University, Jerusalem, Israel; 3 Faculty of Medicine, The Hebrew University, Jerusalem, Israel

**Keywords:** Machine learning, Computational models, Software

## Abstract

Biolord is a deep generative method for disentangling single-cell multi-omic data to known and unknown attributes, including spatial, temporal and disease states, used to reveal the decoupled biological signatures over diverse single-cell modalities and biological systems. By virtually shifting cells across states, biolord generates experimentally inaccessible samples, outperforming state-of-the-art methods in predictions of cellular response to unseen drugs and genetic perturbations. Biolord is available at https://github.com/nitzanlab/biolord.

## Abstract (web summary)

Biolord models the contributions of diverse biological processes to single-cell expression profiles.

## Main

**Fig. 1:**
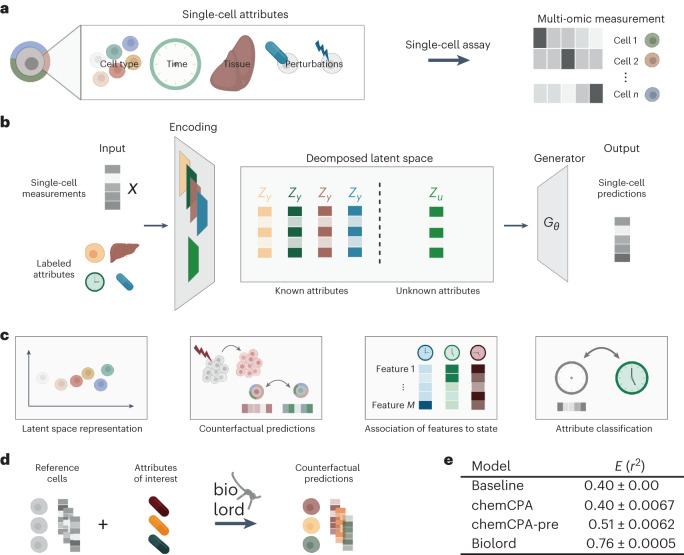
The biolord framework for disentanglement of known and unknown attributes. **a**, Single-cell data encode multiple attributes of cellular identity. **b**, Schematic overview of the biolord model; given single-cell measurements and labels for observed attributes, biolord encodes each attribute separately along with a single encoding of the unknown attributes. These define a decomposed latent space that is the input for the generative module providing measurement predictions. **c**, Biolord can be used for multiple downstream tasks. From left to right—latent space representation: the decomposed latent space can be used to obtain insights into the underlying structure of individual attributes. Counterfactual predictions: given a control cell and unseen (target) labels as input, biolord can predict the gene expression of the unseen cellular states and study the changes in gene expression that correspond to a manipulation of a cell’s attribute. Association of features to state: by manipulating the known attributes, biolord can identify measured features associated with the different possible states, for example, by manipulating control cells to an infected state and identifying genes associated with infection. Attribute classification: using the semi-supervised biolord architecture, cells can be labeled with missing attributes. **d**, Schematic overview for obtaining counterfactual predictions. We take as input measurements of a set of reference cells with varying assignment(s) to the attribute over which predictions are made. For example, we take as input control cells along with multiple drugs that can be applied to generate counterfactual predictions as to how the gene expression profiles of these cells would have been shifted given each drug (Methods). **e**, An evaluation of biolord’s performance on predictions of unseen drugs over the sci-Plex 3 dataset that includes ~650,000 single-cell transcriptomes from three cancer cell lines exposed to 188 compounds^16^. Results are reported for the 10 μM dosage, considered to be the strictest setting since measurements show the largest deviation from control state, which makes them hardest to predict. Mean and variance are reported over ten different random seed initializations of each model. Figure panels **a**–**d** are created with BioRender.com.

A cell’s gene expression profile simultaneously encodes information about multiple attributes, such as cell type, tissue of origin and differentiation stage (Fig. 1a). Single-cell technologies can provide information about such expression profiles for cellular populations at single-cell resolution. Yet, it is still a major challenge to decode the measured gene expression, disentangling the processes from one another. A disentangled representation can uncover the existence and characteristics of diverse biological processes, allowing the reconstruction of multiple attributes of cellular identity such as response to perturbations and infection progression. Earlier studies suggested using factor analysis^1,2^ or non-negative matrix factorization^3^ to identify programs associated with different attributes. Recently, computational methods that specialize in disentanglement for a specific task were suggested; among the addressed tasks are decoupling perturbation response^4–8^, disentangling group-specific attributes^9^ or out-of-distribution sampling of single-cell data^10,11^. However, these are either task-specific and do not address the general disentanglement problem, rely on linearity and independence assumptions, cannot integrate multiple types of information beyond the single-cell measurements or do not provide a generic reconstruction procedure.

In machine learning, disentanglement methods view the world as generated by an unknown forward process that maps the generative factors (attributes) into the observable data. For example, an image of a car is generated by several attributes such as model and pose. The objective of disentanglement is to invert this process, for example, mapping the car image into variables representing its model and pose. The disentangled representation can then be used for data manipulation, generating unseen combinations of model and pose. Analogously, in the biological setting, given labeled single-cell data, for example, cell type and age annotations (known attributes), a disentangled representation will decouple known attributes, cell type and age, from the unknown attributes. The unknown attributes correspond to a cell-specific signature, for example, related to batch effects, biological noise or unclassified biological processes. The disentangled representation can be used for data generation, manipulation and deriving biological insight (for example, predicting the measured features of unobserved combinations of cell type and age or identifying driver genes of certain cell type or state).

**Extended Data Fig. 1:**
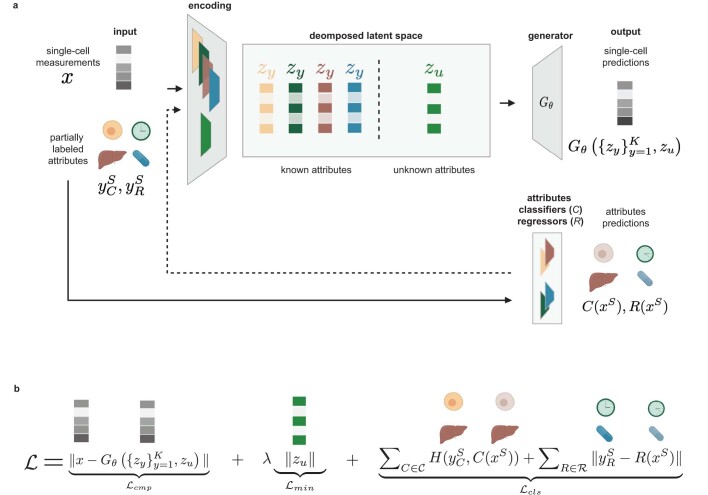
A detailed schematic illustration of biolord. **a**, The semi-supervised biolord architecture, biolord-classify. To handle partial labels we add classifiers to the standard biolord model. The whole framework is optimized jointly (Methods). **b**, An illustration of the biolord loss terms with respect to the different model attributes. Figure panels **a** and **b** are created with BioRender.com.

Using recent advances in disentanglement from the computer vision field^12,13^, we present biolord (biological representation disentanglement), a deep generative framework for learning disentangled representations in single-cell data (Methods). To disentangle single-cell data into its underlying attributes, we assume a training set consisting of single-cell measurements, each with partial supervision over a limited set of known attributes. For example, the known attributes may be cell-type labels, measurement time or perturbation values; attributes may be categorical (discrete; for example, cell type) or ordered (continuous; for example, age). Given the partial supervision, biolord finds a disentangled latent space, consisting of embeddings for each known attribute and an embedding for the remaining unknown attributes in the data (Fig. 1b). On top of these, biolord learns a generator, which maps the representations of the known and unknown attributes into observable single-cell data. It can, in turn, use the disentangled latent space to predict single-cell measurements for different cell states across variations in internal or external conditions. Successful disentanglement is obtained by inducing information constraints; the model’s loss function attempts to maximize the accuracy of the reconstruction (enforcing completeness) while minimizing the information encoded in the unknown attributes (limiting its capacity). We modify the original framework, dedicated to image analysis^12,13^, to account for the features of single-cell data through architecture and design choices (Fig. 1b and Supplementary Note 1; Methods). Furthermore, we present an extension to the framework, biolord-classify, which can be applied to datasets with partially labeled attributes and provides a classification for missing labels (Methods; Extended Data Fig. 1).

The generality of the framework allows its application to diverse biological settings that can be studied with a rich set of downstream analysis tasks (Fig. 1c; Methods). Using the generative aspect of the model, we can make counterfactual predictions, predicting unseen cellular states and performing data manipulation. Applied for the prediction of responses to unseen drugs or gene perturbations, biolord outperforms state-of-the-art methods dedicated to this task. The decomposed latent space representation allows studying the different attributes and their inner structure independently. For example, this representation of the human fetal chromatin atlas revealed the relationships between tissue, sample estimated post-conceptual age and cell-type attributes (Supplementary Notes 1 and 2 and Supplementary Figs. 1–3). Moreover, we can associate measured features with a cell state. At last, biolord can be applied to a partially labeled dataset and used to obtain labeling over the entire dataset (attribute classification). We apply this to a spatiotemporal *Plasmodium* infection atlas to complete the missing classification of a distinct state (initially provided only for the latest time point), thereby allowing us to study the transient trajectory toward the infected state. We implemented biolord using the scvi-tools library^14^ and made it available at https://github.com/nitzanlab/biolord.

## Biolord accurately predicts cellular perturbation response

Accurate prediction of molecular responses to drug or genetic perturbations is central to our understanding of cellular behavior and translational medicine. Hence, many computational tools are dedicated to this task^5–8^^,15^ (Supplementary Note 3). Among these are chemCPA^5^, for drug response prediction, GEARS^6^ for genetic perturbations and PerturbNet^8^, which addresses both (Supplementary Note 3). Cellular response prediction can be framed as a disentanglement task, aimed at decoupling perturbation response from the underlying cell state, and therefore can be approached by biolord. For the drug response prediction task, we use the sci-Plex 3 dataset that includes ~650,000 single-cell transcriptomes from three cancer cell lines exposed to 188 compounds at four different dosages and control samples^16^ (Supplementary Fig. 4).

To allow generalization to unseen drugs, we take advantage of existing prior knowledge and obtain chemically informed embedding of the drugs using RDKit features^5,17^. For each cell, the features of each drug, alongside its dosage, cell line and corresponding scRNA-seq measurements, are given as input to biolord (Methods; Supplementary Note 3). Biolord’s learned latent representation is biologically informative; it reveals drug organization according to known corresponding pathways, and better captures underlying drug organization, relative to the chemically informed RDKit features used as input, both qualitatively and quantitatively (adjusted Rand index RDKit: 0.03, biolord: 0.16; Supplementary Fig. 4). To further evaluate the quality of biolord’s drugs representation, we employ the uncertainty measure suggested by ref. ^5^, assessing the ability to predict the drug’s pathway from the *k*-nearest neighbor (*k*-NN) graph of the embedding space (Methods). Compared to RDKit, biolord’s uncertainty measure is found to be lower on average and more concentrated (distribution evaluated over all drugs; RDKit: 0.32 ± 0.008, biolord: 0.19 ± 0.005; Supplementary Fig. 4).

We use the trained biolord model to obtain counterfactual predictions for nine unseen drugs (reported among the most effective drugs in sci-Plex 3 data^16^, following the choice suggested in ref. ^5^). Specifically, we generate the expression prediction for control cells with labels of unseen compounds. Performance is evaluated using the *r*^2^ score between the real measurements of cells exposed to the unseen compounds and the counterfactual predictions (Fig. 1d; Methods). Biolord outperforms a naive baseline (comparing real measurements of unseen compounds to the control measurements), as well as state-of-the-art models, chemCPA and chemCPA-pre (Fig. 1e, Supplementary Fig. 4 and Supplementary Note 3). Although not provided with the additional information used by chemCPA-pre, biolord provides more accurate predictions (mean *r*^2^; chemCPA-pre: 0.51 ± 0.0062, biolord: 0.76 ± 0.0005). Biolord also outperforms PerturbNet^8^ (Supplementary Note 3) and is robust to data subsampling, retaining high prediction accuracy (mean *r*^2^: 0.63 ± 0.0003) over 10% of the data (Supplementary Fig. 5).

**Extended Data Fig. 2:**
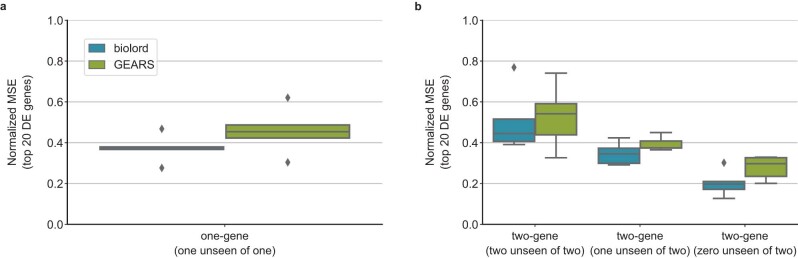
Biolord outperforms GEARS^6^ in predictions of genetic perturbations outcome. **a,b**, The normalized mean squared error (MSE) in the prediction of post genetic perturbation expression of top 20 differentially expressed (DE) genes per perturbation. **a**, The MSE in the prediction of an unseen one-gene perturbation in the Perturb-seq (one-gene) dataset by Adamson et al.^19^. **b**, The MSE in the prediction of two-gene perturbations in the Perturb-seq (two-gene) dataset by Norman et al.^20^ evaluated over perturbations for which: 2, 1 or 0 of the independent two-gene perturbations are unseen. We use the normalization as defined by Roohani et al. in GEARS^6^. This normalization is done with respect to a ‘No perturbation’ model which predicts that there was no effect induced by the perturbation implying that the unperturbed cell state is the same as the post-perturbed one. In all boxplots middle line, median; box boundary, interquartile range (IQR); whiskers, 1.5× IQR; minimum and maximum, not indicated in the box plot; gray dots, points beyond the minimum or maximum whisker.

To demonstrate biolord’s application to the genetic perturbation setting, we consider two genetic perturbation screens that use the Perturb-seq assay^18^. The first is a dataset consisting of 81 one-gene perturbations suggested by ref. ^19^, and the second is a dataset suggested in ref. ^20^ that includes 131 two-gene perturbations and 105 one-gene perturbations. In this setting, to allow for generalization, we use features that are based on edges in a GO term graph defined over genetic perturbations as defined in ref. ^6^ (Methods). We show that biolord outperforms GEARS in the prediction of unseen one-gene perturbation (normalized mean squared error, one of one gene unseen; GEARS: 0.47; biolord: 0.37) and two-gene perturbations (normalized mean squared error, two of two genes unseen; GEARS: 0.53; biolord: 0.50, one of one gene unseen; GEARS: 0.39; biolord: 0.35, zero of two genes unseen; GEARS: 0.28; biolord: 0.20; Methods; Supplementary Note 3 and Extended Data Fig. 2).

## Counterfactual predictions expose infection gene programs

**Fig. 2:**
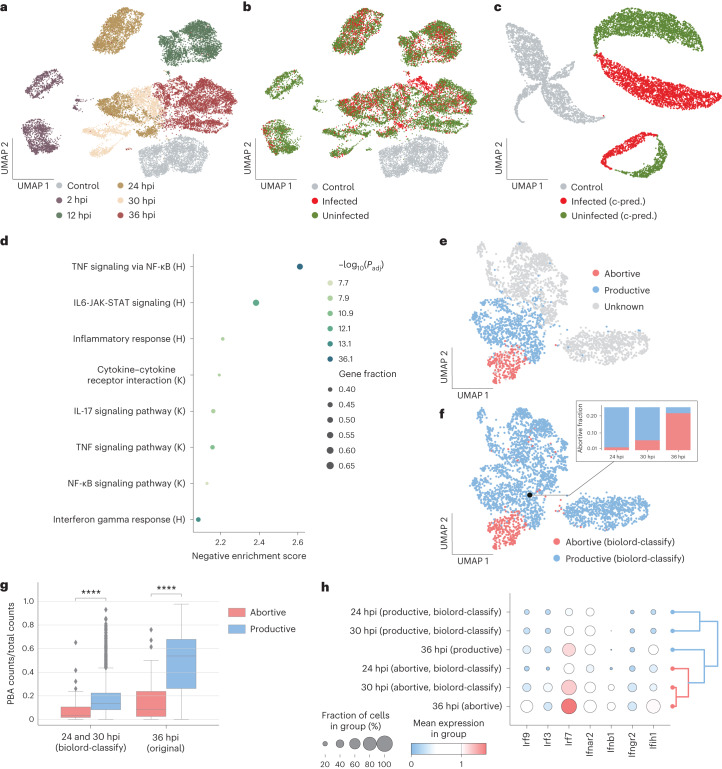
Recovering transient states by classifying unknown cell states using biolord. **a**,**b**, UMAPs of the single-cell atlas of the *Plasmodium* liver stage^21^. Cells are colored by time after infection (**a**) and reported classification to infected/uninfected and control cells (**b**). **c**, UMAP of the original control cells with their counterfactual predictions (c-pred.) for infected/uninfected state; cells are colored by the corresponding state. **d**, GSEA of genes found to be associated with the infected state based on biolord’s counterfactual predictions of the infection state in control cells. H denotes Hallmark gene sets; K denotes KEGG gene sets (*P*_adj_ is calculated using a permutation test with Benjamini–Hochberg correction). **e**,**f**, UMAPs of the infected cells from intermediate to late time points in the single-cell atlas of the *Plasmodium* liver stage^21^. Cells are colored by the reported abortive/productive classification of cells at 36 hpi (**e**) and biolord’s classification of all infected cells as abortive/productive (**f**). The inset shows the fraction of abortive cells at each time point (24 hpi, 0.016; 30 hpi, 0.057 and 36 hpi, 0.215). **g**, Box plot comparing abortive and productive cells shows that abortive hepatocytes retain a smaller fraction of *Plasmodium* transcriptome across all time points. Middle line in box plot, median; box boundary, IQR; whiskers, 1.5× IQR; minimum and maximum, not indicated in the box plot; gray dots, points beyond the minimum or maximum whisker (Mann–Whitney–Wilcoxon test two-sided with Benjamini–Hochberg correction: 24 and 30 hpi (*n* = 1,823 cells across two states); biolord-classify < 0.0001, 36 hpi (*n* = 1,083 cells across two states); original < 0.0001; ****P ≤ 0.0001). **h**, Abortive cells present an over-expression of interferon response as demonstrated by an increase in interferon regulatory factors and response-associated genes. The dendrogram ordering of the groups shows a trajectory from productive to abortive cells ordered by hpi. IQR, interquartile range.

**Extended Data Fig. 3:**
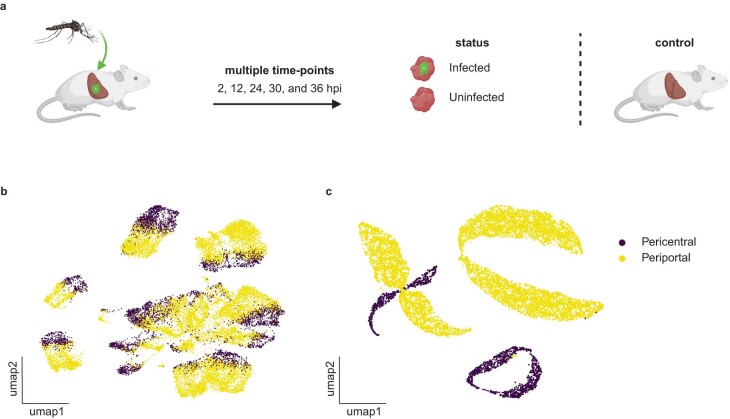
Biolord applied to the single-cell atlas of the *Plasmodium* liver stage^21^. **a**, Experimental schematic. GFP+ parasites are injected into mice and liver samples are extracted at different time points. Hepatocytes are classified as infected/uninfected using FACS sorting. Control samples are collected from healthy mice. Figure is created with BioRender.com. **b**, UMAP of the single-cell atlas of the Plasmodium liver stage; cells are colored by spatial zone. **c**, UMAP of the original control cells with their counterfactual predictions (c-pred.) for infected/uninfected state; cells are colored by spatial zone.

The collection of spatiotemporal single-cell atlases is continuously expanding, each capturing a complex biological setting. Among the computational challenges is disentangling the diverse attributes, thereby associating the measured features with distinct cell states. Focusing on a spatiotemporal single-cell atlas of *Plasmodium* infection progression in the mouse liver^21^, we show that biolord can obtain a disentangled representation that allows for uncovering infection-related attributes. Single-cell data, including host and parasite transcriptome, were collected from infected mice at five time points post-infection (2, 12, 24, 30 and 36 h post-infection (hpi)), as well as from control mice, not exposed to the parasite (control; Fig. 2a and Extended Data Fig. 3). To classify hepatocytes as infected or uninfected, the authors relied on GFP content in the parasite transcriptome^21^ (Fig. 2b). Using biolord, we aimed at decoupling the changes in gene expression in the host hepatocytes induced by the infection from the variability rooted in previously established spatiotemporal processes^22,23^—either in spatial zonation across liver lobules radial axis or in temporal variation along the time of day (Fig. 2a and Extended Data Fig. 3).

We train a biolord model with hepatocytes from injected mice (infected and uninfected) and control mice, along with additional known attributes; namely providing as input the host transcriptome, status classification (infected/uninfected/control), spatial zone (periportal/pericentral) and time (2, 12, 24, 30, 36 hpi or control; Fig. 2c and Supplementary Note 4; Methods). The model is then used to make counterfactual predictions over the population of control cells coupled to infected status. Since the status (infected) is the only attribute modified in the input to the biolord model, for a given cell, observed changes in gene expression are driven only by this attribute (Methods).

To assess these infection-related changes at the level of individual cells, we use a dependent *t* test for paired samples. The pairs are defined as the original observed expression and the infected state counterfactual prediction. We performed the test for each gene and used the results as input for gene set enrichment analysis (GSEA), which revealed an increase in the expression of genes associated with immune and stress pathways in infected hepatocytes (Fig. 2d). These findings are in accordance with previous reports^21^. However, in the original analysis, the comparisons between infected and uninfected hepatocytes had to be done for cells that were matched in terms of spatial lobule coordinates and sampling time. As described above, using biolord’s counterfactual predictions over control cells allowed for global integrated analysis.

## Exposing transient trajectories toward infection states

**Extended Data Fig. 4:**
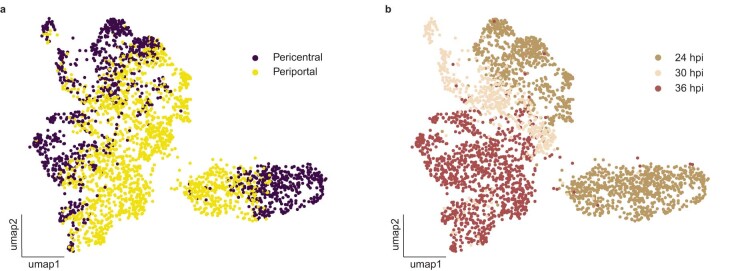
Application of biolord-classify to late time points of *Plasmodium* infection. **a**,**b**, UMAP of cells from late time points, 24, 30, and 36 hours post infection (hpi); cells colored by spatial zone (**a**) or hpi (**b**).

So far, we have assumed full supervision over known attributes (for a known attribute, all cells are labeled); however, this is not always the case. Often only a subset of the cells is annotated. In such cases, we can leverage these partial labels to classify the remaining cells using biolord-classify, a biolord model coupled with a classifier for each attribute, used for the prediction of missing labels (Extended Data Fig. 1 and Supplementary Note 1; Methods). The spatiotemporal single-cell atlas for *Plasmodium* infection^21^, presented above, provides an example of such a setting. Afriat et al.^21^ identified a subpopulation of cells that shows a pattern of vacuole breakdown, termed ‘abortive hepatocytes’. In the scRNA-seq data, this population was identified only at the latest time point (36 hpi; Fig. 2e and Extended Data Fig. 4). However, in analyzing smFISH images, the existence of this population was verified as early as 24 hpi^21^. This motivated to use biolord-classify to classify abortive cells within the scRNA-seq data at earlier time points or, in other words, identify the cells that would have progressed to become identifiably abortive at 36 hpi.

We train a biolord-classify model over hepatocytes at late time points (24, 30 and 36 hpi) using the host transcriptome along with partial state classification and complete supervision over spatial zone and time as inputs (Supplementary Notes 1 and 4). The biolord-classify model is used to label cells at earlier time points (24 and 30 hpi) as abortive or productive, thus predicting a temporally extended abortive population (Fig. 2f; Methods).

**Extended Data Fig. 5:**
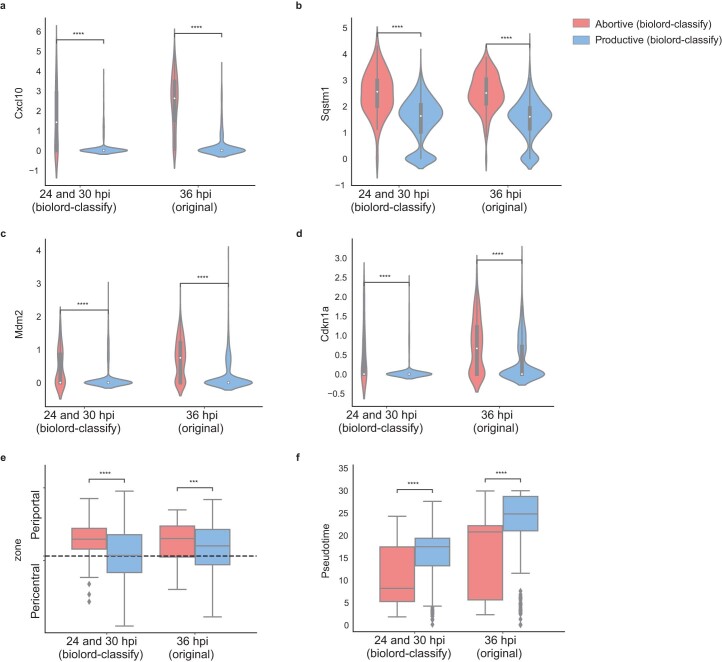
Gene expression patterns are recovered in abortive hepatocytes identified by biolord-classify. **a**–**d**, Violin plots of representative genes upregulated in abortive hepatocytes. Mann-Whitney-Wilcoxon test two-sided with Benjamini-Hochberg correction P-values. *Cxcl10*; 24 and 30 hpi (biolord-classify) < 0.0001, 36 hpi (original) < 0.0001 (**a**), *Sqstm1*; 24 and 30 hpi (biolord-classify) < 0.0001, 36 hpi (original) < 0.0001 (**b**), *Mdm2*; 24 and 30 hpi (biolord-classify) < 0.0001, 36 hpi (original) < 0.0001 (**c**), *Cdkn1a*; 24 and 30 hpi (biolord-classify) < 0.0001, 36 hpi (original) < 0.0001 (**d**). **e**, Boxplots comparing abortive and productive cells show that in accordance with the original abortive hepatocytes population, biolord classified abortive hepatocytes are more periportally zonated compared with productive hepatocytes; the y-axis represents zonation score and scores corresponding to Periportal/Pericentral spatial zones are indicated (Methods, Mann-Whitney-Wilcoxon test two-sided with Benjamini-Hochberg correction P-values: 24 and 30 hpi (biolord-classify) < 0.0001, 36 hpi (original) < 0.0001. **f**, The abortive population is concentrated at early pseudotime. Pseudotime was evaluated over parasite mRNA^21^ (Mann-Whitney-Wilcoxon test two-sided with Benjamini-Hochberg correction P-values: 24 and 30 hpi (biolord-classify) < 0.0001, 36 hpi (original) < 0.0001). All statistical tests at 24 and 30 hpi are reported for n = 1,823 cells across two states; and at 36 hpi for n = 1,083 cells across two states. In all plots middle line, median; box boundary, interquartile range (IQR); whiskers, 1.5× IQR; minimum and maximum, not indicated. In (a)-(d) the symmetric kernel density estimate is shown and in (e)-(f) gray dots, points beyond the minimum or maximum whisker. ***P ≤ 0.001, ****P ≤ 0.0001.

The extended abortive population preserves host gene expression trends observed in the original 36 hpi population. Namely, representative genes found to be upregulated in abortive hepatocytes at 36 hpi^21^ are statistically significantly upregulated in predicted abortive cells across all time points (Extended Data Fig. 5). Furthermore, cells predicted to be abortive by biolord show reduced levels of *Plasmodium* transcripts and appear at earlier phases of *Plasmodium-*based pseudotime, consistent with findings regarding the original abortive population at 36 hpi^21^, although these attributes were not used to train the biolord-classify model (Fig. 2g and Extended Data Fig. 5; Methods). Additionally, we recover the periportal bias of the abortive population^21^ in the newly classified abortive cells (Extended Data Fig. 5).

The increased IFN response across all time points, demonstrated by the over-expression of interferon regulatory factors (*Irf3*/*Irf7*/*Irf9*), which regulate the transcription of type I IFNs, and an increase in IFN*α*, IFN*γ* genes demonstrated by the extended abortive population are consistent with the hypothesis linking the abortive state to interferon-mediated innate immune response induced by the *Plasmodium* liver stage (Fig. 2h)^21,24,25^. Furthermore, biolord captures a transient trajectory of cellular states, showing a gradual increase in IFN signal across time within the abortive subpopulation (Fig. 2h).

## Discussion

To summarize, we demonstrated biolord’s application to a wide variety of tasks, considering diverse single-cell modalities and biological systems, showcasing the range of insights such disentangled representations can provide.

While here we focused on disentangled representations with respect to known attributes, an intriguing follow-up direction is to study the representation of unknown attributes. In addition, similar to other disentanglement methods, it is unclear what is the desired outcome when attributes are correlated. This will not adversely affect the result when aiming to predict previously seen combinations (for example, if measurements of cell type *X* in tissue *Y* are provided). However, predictions over unseen combinations may yield unpredictable results, which is a known limitation of neural networks. With that, by providing a decomposed latent space, biolord allows extracting the underlying structure of each biological attribute independently, mitigating the above limitations. At last, as with any deep generative framework, biolord suffers from the lack of direct interpretability. We overcome this by suggesting various downstream analysis tools, using both the decoupled latent embedding and the generative model, providing biological insight and interpretability in feature (for example, gene expression) space.

To conclude, biolord provides a step toward decoupling cellular identities encoded in single-cell data. It elucidates the effects of the different components on the overall observed expression, thereby providing new insights and better utilization of multi-omic data.

## Methods

### Latent optimization as an inductive bias in disentanglement

Latent optimization is a critical component of our approach. Typical representation disentanglement approaches use an encoder to map the original data samples into latent codes. This is often called amortized inference. While having an encoder network to map samples to codes is convenient, Gabbay and Hoshen^12^ showed that this approach may achieve subpar results. The reason is that at the beginning of training, an encoder (which is randomly initialized) maps all sample attributes to each latent code, both known and unknown. While the loss function encourages disentanglement (removal of the known attribute), the random initialization of the encoder causes the optimization to begin from a perfectly entangled state. Later training iterations struggle to remove this entanglement entirely.

In contrast, randomly initialized latent codes trivially do not contain any information on known or unknown attributes. While training, the latent code corresponding to each sample becomes more informative over the unknown attribute, while the disentanglement objectives ensure that it does not gain information over the known attribute. Intuitively, preventing the gain of unwanted information is easier than losing existing information. To conclude, latent optimization helps achieve more disentangled latent codes by providing a better initialization for the learning process.

It is important to note that the results obtained in ref. ^12^ directly apply to the biological setting presented here. As detailed above, the challenge, which is resolved by the latent optimization, is with respect to the labeled attributes. As these are the labels provided along with the sample (image or single-cell measurements), they are identical in both settings. Hence, the latent optimization allows us to obtain meaningful latent codes with respect to the target attributes.

### The biolord model

Biolord is a deep learning generative framework, composed of multiple modules that are jointly optimized. The input to biolord is a dataset of $D={\left[\left({x}_{c},{y}_{c}\right)\right]}_{c=1}^{n}$, where *n* is the number of cells. For each cell *c*, ${x}_{c}\in {{\mathbb{R}}}^{M}$ stands for the *M* measured features (for example, a vector of gene expression counts or peak counts from *M* genes), and *y*_*c*_ is a set of size *K* representing the known cell attributes, for example, cell-type label, tissue of origin or age. As we elaborate below, within *y*_*c*_, we make a distinction between categorical and ordered attributes when constructing its corresponding latent space. In accordance, each of the *K* elements in the set *y*_*c*_ may be of a different dimension. Given the input dataset *D*, the biolord pipeline consists of two main components, defined and trained simultaneously (construction details are provided in the following subsections and Supplementary Note 1):

1. Decomposed latent space—for each known attribute, a dedicated subnetwork is constructed. The architecture of each subnetwork is chosen based on the attributes’ type (categorical or ordered), and the user can modify additional hyperparameters. We denote *z*_*y*_ as the output of each subnetwork, which is the latent space corresponding to an attribute (categorical or ordered) in *y*_*c*_, and *z*_*u*_ as the latent space of unknown attributes (Fig. 1b).
2. Generative module—the generator *G* takes the concatenated decomposed latent space as input and outputs a prediction for the measured features.

It is important to note that the optimization of the above, the decomposed latent space and the generative prediction, is done jointly, such that the embeddings in the decomposed latent space are optimized with respect to the reconstruction error of the generator.

#### Known attributes latent space

Given the known attribute set, *y*_*c*_, a dedicated subnetwork is constructed for each of the *K* attributes to represent its corresponding latent space. Here we make a distinction between categorical attributes, where similar cells share class labels, and ordered attributes, in which distances between the attribute’s features encode similarity. In our definition of ordered attributes, we consider continuous variables as well as categorical ordinal variables, as the important aspect is that attribute’s features contain structural information. Furthermore, measured categorical ordinal variables (such as age) often represent a sample of continuous variables. With this, we construct the different subnetworks as follows:

- Categorical attribute subnetworks—these are defined using the embedding module such that the latent code, *z*_*y*_, is shared between all cells belonging to the same label. The embeddings are optimized directly, namely applying latent optimization through the objective function of the complete model.
- Ordered attribute subnetworks—to use the structure of each of the ordered attributes, we use encoders; multilayer perceptrons (MLPs) with default values of depth = 2, width = 256. The MLPs map the input features to a corresponding latent space, *z*_*y*_, which is optimized using the objective function of the complete model.

#### Unknown attributes latent embedding

We learn the unknown attributes’ representation by optimizing per-sample embeddings directly. We use a regularized embedding subnetwork, an embedding module to which Gaussian noise, *η*, a random variable $\eta \sim {\mathscr{N}}(0,{\sigma }^{2}I\,)$, with a fixed variance value *σ*, is added (Supplementary Note 1). The output is a unique latent code, *z*_*u*_, for each cell, independent of gene expression or known attributes, optimized during training using latent optimization.

With that, optimizing a unique code for each cell may hinder our disentanglement efforts; the model may encode the entire expression information with the latent code of unknown attributes and ignore the attribute-specific encoding. Following Gabbay and Hoshen^12^, to ensure that known attribute information does not leak into the representation of the unknown attributes, we regularize it into two manners. First, we introduce the additive Gaussian noise to the embeddings, and second, we add an activation penalty term to the loss, limiting the magnitude of the embedding, thus inducing the minimality loss term,

$${ {\mathcal L} }_{\rm{min}}=\lambda {\Vert {z}_{u}\Vert }^{2},$$

where *λ* is a hyperparameter weighting this term. Together, these enforce the minimality of shared information between the representation of unknown attributes and known attributes. That is, the representation of unknown attributes is optimized to minimize the information it encompasses regarding known attributes.

#### Generator module

The generator *G* is constructed as a decoder network, parameterized by *θ*, which takes as input the concatenated decomposed latent space, and outputs a parametrization of the expression distribution of the measured features (given by the mean and variance),

$$P={G}_{\theta }\left({\left\{{z}_{y}\right\}}_{y=1}^{K},{z}_{u}+\eta \right).$$

Depending on the data provided as input to the model, preprocessed log-normalized data, raw counts or peaks, the distribution, *P*, can follow a Gaussian distribution, a zero-inflated negative binomial or Poisson, respectively^14,26^. To define the reconstruction, and completeness loss term, we use the respective negative log-likelihood loss for each distribution, ${\rm{NLL}}\left(x|{G}_{\theta }\right)$. Following the original model presented by Gabbay and Hoshen^12^, we include a mean squared error term, concerning the predicted means, *μ*_*θ*_, as provided by, *G*_*θ*_, ${\rm{MSE}}\left(x,{\mu }_{\theta }\right)$, tuned by *τ* (‘reconstruction_loss’) hyperparameter. This allows us to directly optimize the mean predictions, for all choices of parametric distribution modeling (Supplementary Note 1). Hence we can write the completeness term as,

$${{\mathcal{L}}}_{{\rm{cmp}}}={\rm{NLL}}\left(x|{G}_{\theta }\right)+\tau {\rm{MSE}}\left(x,{\mu }_{\theta }\right).$$

#### Model optimization

Combining the above, we can write the complete model objective as a composition of two terms. The first term induces completeness by optimizing the accuracy of the generator, and the second term enforces the minimality of information shared between the representations of known and unknown attributes,

$${{\mathcal{L}}}_{{\rm{biolord}}}={{\mathcal{L}}}_{{\rm{cmp}}}+{{\mathcal{L}}}_{\min }$$

Since the different components defined above are jointly optimized, the embeddings within the decomposed latent space along with the generator’s predicted output are influenced by input measurements as well as the known attribute labels.

### Biolord-classify: biological representation disentanglement with partial labels

To perform semi-supervised disentanglement, a setting in which we have missing labels for a subset of cells, we adopt the derivation presented in ref. ^13^. In addition to the biolord model components described above (the decomposed latent space and generative module), we include a classifier, $C\in {\mathcal{C}}$, for each categorical attribute, and a regressor, $R\in {\mathcal{R}}$, for each ordered attribute, which are trained together with previous components.

The classifier (regressor) takes as input the gene expression and outputs the class label/features. For cells with missing labels, the classifier’s (regressors) output is used to complete the decomposed latent representation (Extended Data Fig. 1). To train the classifiers (regressors), we add a term to the existing loss function that encourages the correct prediction for the samples for which labels are available. For the classifiers, we use the categorical cross-entropy loss, $H\left(y,C\left(x\right)\right)$. For the regressors, we use the mean squared error loss between the output and provided features, ${\rm{MSE}}\left(\,y,R\left(x\right)\right)$. In all cases, the loss is evaluated only over cells for which labels are provided (denoted by the sets ${X}^{\,S},{Y}^{\,S}$). The classification loss is then provided by,

$${{\mathcal{L}}}_{{\rm{cls}}}=\sum _{C{\mathscr{\in }}{\mathcal{C}}}H\left({Y}_{C}^{\,S},C\left({X}^{\,S}\right)\right)+\sum _{R{\mathscr{\in }}{\mathcal{R}}}{\rm{MSE}}\left({Y}_{R}^{\,S},R\left({X}^{\,S}\right)\right),$$

where ${Y}_{C}^{\,S}\left({Y}_{R}^{\,S}\right)$ denotes the set of labels (features) associated with the respective classifier, *C* (regressor, *R*). ${{\mathcal{L}}}_{{\rm{cls}}}$ is added to the biolord objective, such that all components are now trained jointly,

$${{\mathcal{L}}}_{{\rm{biolord}}-{\rm{classify}}}={{\mathcal{L}}}_{{\rm{cmp}}}+{{\mathcal{L}}}_{\min }+{{\mathcal{L}}}_{{\rm{cls}}}.$$

By including the classification module (classifiers and regressors) as part of the biolord training procedure, we allow training of a biolord model in a semi-supervised setting, since the classifiers and regressors are used to impute missing labels used as input for the decomposed latent cells. Furthermore, the imputed labels can be used in downstream analysis of the data (Fig. 1c).

### Biolord-enabled downstream analysis

Biolord enables diverse downstream analysis tasks using the decoupled latent representation, the generative pipeline and the biolord-classify module (Fig. 1c). Within the biolord framework, we provide utility functions to enable this analysis. The downstream tasks are given as follows:

1. Latent space representation—the latent space embeddings provide insight into the structure within a specific attribute and between the different attributes. The latent representation is a set of vectors mapping the states of the known attributes to a *n*_latent_ dimensional state. Any downstream analysis tool can be now applied to expose properties and relationships between the states, for example, correlation analysis, clustering or lower dimensional representation. The latent representation can be used to explore structure between different labels of a given attribute, for example, using a correlation matrix, or to study interactions between the different attributes by considering a concatenated representation.
2. Uncertainty evaluation—uncertainty measures provide a proxy to assess the generalizability of a model. We use an evaluation metric presented in ref. ^5^ which allows quantifying the uncertainty of an attribute over its latent representation when additional covariates associated with the attribute are known, for example, pathway association of the drug attribute, and provide its implementation in the biolord package. The uncertainty is defined by the inability to predict the covariate (the drug’s pathway) from the *k*-NN graph of the attribute’s latent space representation. Formally, we define,
  $${u}_{i}=\sum _{j\in {{\mathscr{N}}}_{i}}\frac{1}{\log d\left(i,j\right)}\times H\left({C}_{{{\mathscr{N}}}_{i}}\right),$$
  where ${{\mathscr{N}}}_{i}$ is the set of neighbors of value *i*, *d* is a distance measure and *H* is the Shannon entropy, and ${C}_{{{\mathscr{N}}}_{i}}$ is the covariate vector associated with neighbors of *i* based on the latent representation.
3. Counterfactual predictions—the biolord module can take a specific cell instance and modify its known attributes. The unknown attribute embedding obtained by biolord captures a cell-specific embedding. Hence, when passing as input the measured features of a cell along with different labels for known attribute(s) of interest, the cell-specific attributes representation will remain constant (the unknown attribute embedding) and only the embeddings of modified known attributes will change. Since the embeddings are the input for the generative module providing the predictions, all observed changes are induced by the modification of the known attribute(s). To obtain counterfactual prediction in practice, we take a set of reference cells, for example, control cells in an infection dataset, and use their measured features along with any combination of known attributes (for example, modify the state label considering infected case) as input to a trained biolord model. This allows us to first sample unseen biological states and more importantly obtain a controlled set of samples where we are guaranteed that all observed changes in the measured features are a result of the modified attribute (Fig. 1d).
4. Association of features to state—pairing the counterfactual predictions with a statistical test allows us to recover a set of features (for example, genes) that encode the given observed state. Here we explicitly decouple the modified attribute from the underlying cell state; hence, observed changes in the predictions are induced by the modified attributes.
5. Classification—the biolord-classify module can extend the labeling of partially labeled attributes. This provides complete labeling of the data that can in principle be further inspected and used as input for diverse downstream analysis pipelines.

### Datasets, training and evaluation

#### Sci-Plex 3

The sci-Plex 3 dataset^16^ contains measurements for 649,340 cells across 7,561 genes from three human cancer cell lines—A549, MCF7 and K562 with perturbations for 188 drugs at four different dosages, 10 nM, 100 nM, 1 μM and 10 μM. We use a preprocessed anndata file provided in ref. ^5^, downloaded from https://f003.backblazeb2.com/file/chemCPA-datasets/sciplex_complete_middle_subset.h5ad. To the downloaded anndata file, we add RDKit features^17^ using chemprop package^27^ and an out-of-distribution split, keeping nine unseen drugs for validation—Dacinostat, Givinostat, Belinostat, Hesperadin, Quisinostat, Alvespimycin, Tanespimycin, TAK-901 and Flavopiridol.

##### Training parameters

We train a biolord model over the processed gene expression. We use RDKit chemically informed features embedding of the drugs^17^, as well as the dosage as ordered attributes. The cell line is passed as a categorical attribute. We used Weights & Biases^28^ for experiment tracking and hyperparameter tuning. Hyperparameter details are provided in Supplementary Note 3.

##### Evaluation and benchmarks

Following the setting provided by ref. ^5^, we evaluate the prediction accuracy using the coefficient of determination *r*^2^ (*r*^2^ score), calculated between a model’s counterfactual predictions and the ground-truth measurements on all genes.

The included benchmarks were as follows:

1. naive baseline—the *r*^2^ score is evaluated between control, unperturbed cells (per cell line) and the respective drug-treated cells.
2. chemCPA^5^—the standalone setting that trains the drug encoding network directly on the single-cell data using reported optimal hyperparameters^5^.
3. chemCPA-pre^5^—a pretrained model, for which the drug encoding network was trained over bulk RNA high-throughput screen (L1000)^29^. The pretrained model was kindly shared with us by the authors of chemCPA^5^. Hyperparameter tuning for all adversary parameters was performed.
4. PerturbNet^8^—the model consists of three networks, a perturbation representation network, a cellular representation network and a mapping network. For the perturbation representation network, we use the pretrained model provided by ref. ^8^ trained on the ZINC dataset^30^. The remaining networks were trained following the example provided in the PerturbNet online Github repository. The cellular representation network was trained over the anndata file described above. The mapping network was trained over the latent representation provided by both trained models.

Further details regarding all frameworks are provided in Supplementary Note 3.

#### Genetic perturbations

##### Perturb-seq (one-gene)

The Perturb-seq dataset^19^ contains measurements of 65,899 cells across 5,060 genes, including 81 one-gene perturbations and control cells. We use the preprocessed anndata provided by GEARS^6^. To obtain meaningful features (representing the genetic perturbations), we use the perturbation edges in the GEARS’ Gene Ontology (GO) graph. The GO graph was originally generated by adding weighted edges between genes that share a significant number of GO terms^6^. Lastly, for training, we consider only the averaged expression over each perturbation and the control cells.

##### Perturb-seq (two-gene)

The Perturb-seq dataset^20^ contains measurements of 89,357 cells across 5,045 genes, including 131 two-gene perturbations, 105 one-gene perturbations and control cells. We use the preprocessed anndata object provided by GEARS^6^. As above, we leverage the GEARS’ GO graph to obtain meaningful features that represent the genetic perturbations. For training, we consider only the averaged expression over one-gene perturbations and the control cells. To obtain predictions over the two-gene perturbations, we approximate the difference in expression as the sum of the difference in prediction of each one-gene perturbation.

##### Training parameters

We train a biolord model using the mean expression for perturbation in the train set. We follow the setting defined in GEARS that considers five different train-test-validation splits differing in the set of unseen perturbations. For the two-gene perturbation setting, we make the distinction in one of five splits between perturbations for which two, one or zero of the two-gene perturbations are unseen during training. We use the GO term features as an ordered attribute for the model. We used Weights & Biases^28^ for experiment tracking and hyperparameter tuning (Supplementary Note 3).

##### Evaluation and benchmarks

Following the procedure suggested in ref. ^6^, we evaluate the normalized mean squared error in the prediction of unseen perturbations. Normalization is done with respect to predictions in a ‘no perturb’ setting, that is predictions that there was no effect of performing a perturbation; hence, the unperturbed cell state is the same as the post-perturbed one.

For benchmarking, we compare our performance to GEARS^6^, running the evaluation using the setting provided in their reproducibility repository (https://github.com/yhr91/gears_misc/blob/main/paper/fig2_train.py).

#### Spatiotemporal single-cell atlas of the *Plasmodium* liver stage

To study the liver stage of the malaria parasite *Plasmodium*, Afriat et al.^21^ molecularly characterized thousands of infected and uninfected hepatocytes at five time points post-infection (2, 12, 24, 30 and 36). We downloaded the preprocessed annotated data provided by the authors from Zenodo^31^. The data annotations include the following:

- coarse_time: denoting the number of hpi when the cells were collected (or control).
- eta_normalized: a spatial zonation score based on zonation marker genes which were used to classify the cells as periportal/pericentral.
- pseudotime: calculated using Monocle over the normalized data of the infected hepatocyte PBA genes subset.
- status: infection status inferred by FACS sorting of the hepatocytes.
- abortive: classification of cells at 36 hpi as abortive/productive based on clustering of host transcriptome.

##### Training parameters

We define two biolord settings, as described below. Hyperparameters for reported results are provided in Supplementary Note 4.

##### Infected state analysis over the complete dataset

A biolord model is defined over hepatocytes from injected mice (infected and uninfected), as well as control mice (the dataset excluding mock and mosquito bitten samples). As input, we use the host transcriptome (restricted to 8,355 genes used in the original publication) along with status classification (infected/uninfected/control), spatial zone (periportal/pericentral) and time (2, 12, 24, 30, 36 hpi or control).

##### Abortive state classification

A biolord-classify model was trained over infected hepatocytes at 24, 30 and 36 hpi. The host transcriptome (restricted to highly variable genes) along with spatial zone (periportal/pericentral), time (24, 30 and 36 hpi), a stress_score (computed using scanpy’s^32^ function ‘scanpy.tl.score_genes()’ with stress genes^21^) and the partial abortive state classification for 36 hpi (abortive/productive). We introduce the stress_score to disentangle the stress signal, reported in the original publication^21^, from the abortive signature.

### Reporting summary

Further information on research design is available in the Nature Portfolio Reporting Summary linked to this article.

## Online content

Any methods, additional references, Nature Portfolio reporting summaries, source data, extended data, supplementary information, acknowledgements, peer review information; details of author contributions and competing interests; and statements of data and code availability are available at 10.1038/s41587-023-02079-x.

## Supplementary information

Supplementary Information Supplementary Figs. 1–5, Notes 1–4 and Tables 1–16.

Reporting Summary

## Data Availability

Datasets analyzed in this manuscript are publicly available. Processed data files can be downloaded from figshare (https://figshare.com/projects/biolord_datasets/160085). The original datasets analyzed in the current study are available at • Sci-Plex3: https://f003.backblazeb2.com/file/chemCPA-datasets/sciplex_complete_middle_subset.h5ad, a preprocessed file provided by ref. ^5^. • Perturb-seq (one-gene): https://dataverse.harvard.edu/api/access/datafile/6154020, preprocessed data and additional files provided by ref. ^6^. • Perturb-seq (two-gene): https://dataverse.harvard.edu/api/access/datafile/6894431, preprocessed data and additional files provided by ref. ^6^. • Fetal chromatin accessibility atlas: 10.6084/m9.figshare.24886248.v1, a preprocessed file provided by ref. ^33^. • Spatiotemporal single-cell atlas of the *Plasmodium* liver stage: publicly available at GSE181725 or as processed Seurat object at https://zenodo.org/record/7081863.

## Code availability

We implemented biolord using the scvi-tools library^14^ and using cookiecutter-scverse (https://github.com/scverse/cookiecutter-scverse) as a template for the package. The package is released as open-source software at https://github.com/nitzanlab/biolord. Documentation is available at https://biolord.readthedocs.io. The code to reproduce the results is available at https://github.com/nitzanlab/biolord_reproducibility.
