# Supplementary material for: Disentanglement of single-cell data with biolord
Source: Nat Biotechnol. 2024 Jan 15;42(11):1678–83. doi: 10.1038/s41587-023-02079-x (PMC11554562; doi:10.1038/s41587-023-02079-x)
Supplement: Supplementary file 1 — Supplementary Figs. 1–5, Notes 1–4 and Tables 1–16. [file 41587_2023_2079_MOESM1_ESM.pdf]

nature biotechnology

Brief Communication https://doi.org/10.1038/s41587-023-02079-x

Disentanglement of single-cell data with biolord

In the format provided by the authors and unedited

Contents

Supplementary Figures 2

Supplementary Note 1 7

The biolord framework 7
Training a biolord model 8
Training a biolord-classify model 9
Applying biolord to chromatin accessibility atlas 10

Supplementary Note 2 12

Fetal chromatin accessibility atlas 12

Supplementary Note 3 14

Predicting cellular responses to perturbations 14
sci-Plex 3 15
Perturb-seq (one-gene) 20
Perturb-seq (two-gene) 22

Supplementary Note 4 24

Spatio-temporal single-cell atlas of the Plasmodium liver stage 24

Supplementary Figures

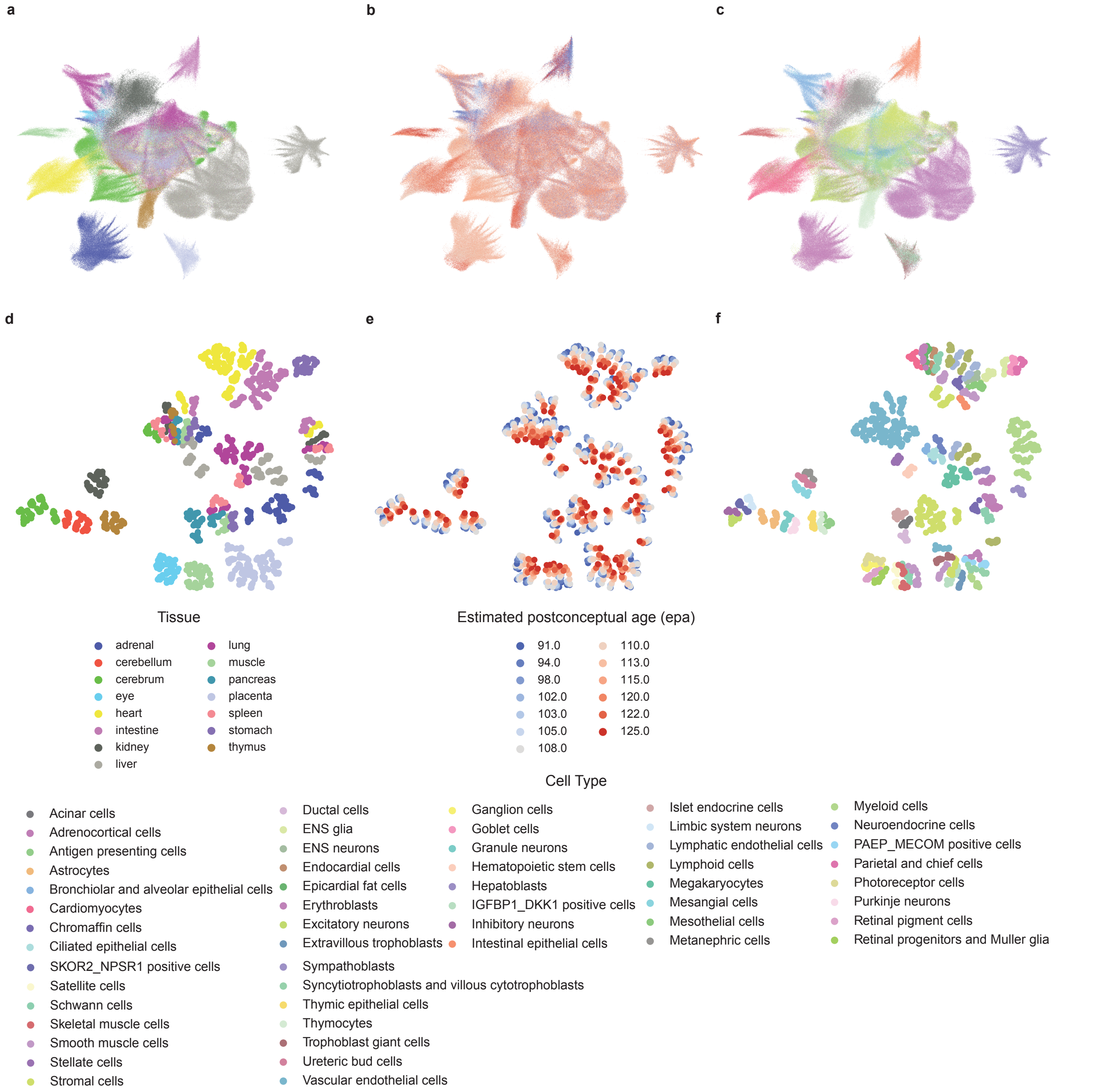

Supplementary Figure 1: Application of biolord to the human fetal chromatin accessibility atlas[1]. a–f, UMAPs of the original data (top row) and biolord's latent embedding (bottom row) of the fetal chromatin accessibility atlas[1]. Biolord's latent representation depicts the concatenated latent embedding space, such that each point represents a combination of tissue, epa and cell type. From left to right cells are colored by **(a)**,**(d)** tissue, **(b)**,**(e)** epa, and **(c)**,**(f)** cell type.

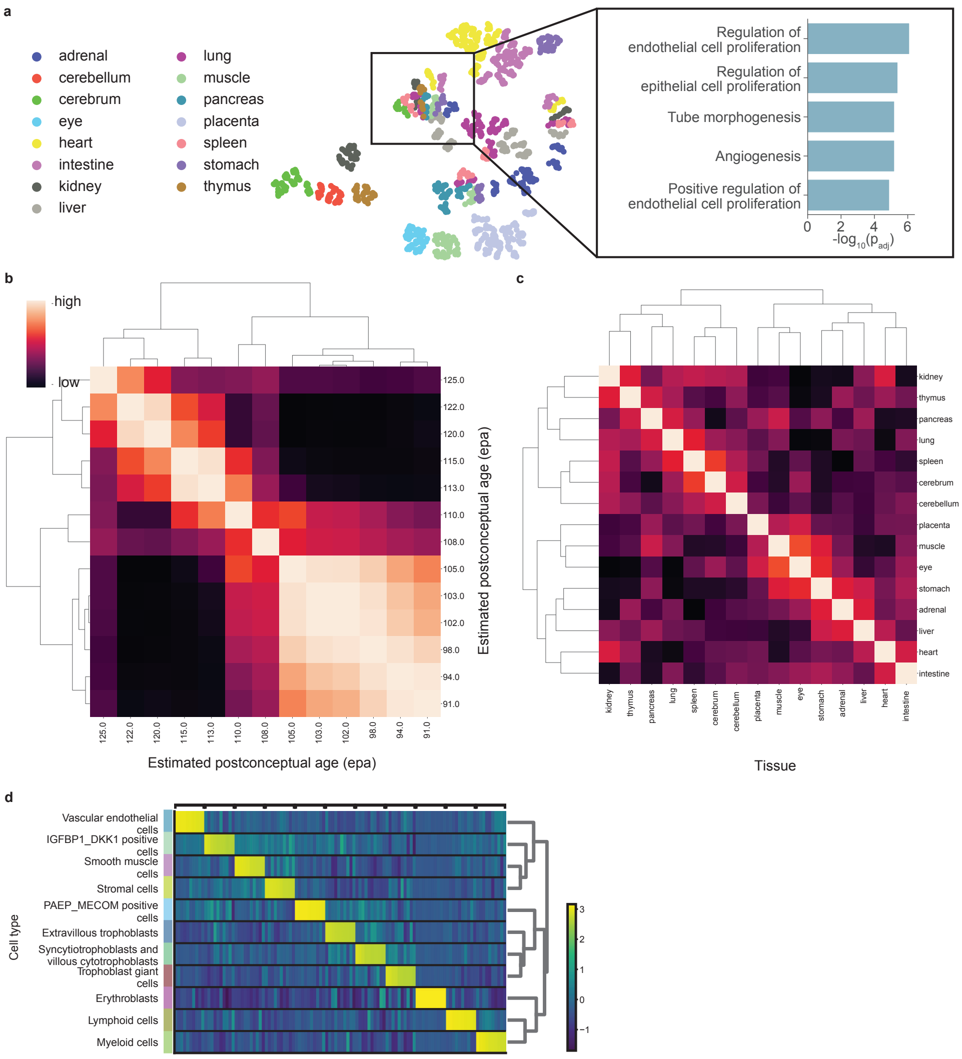

Supplementary Figure 2: Biolord exposes distinct attributes in the landscape of the human fetal chromatin accessibility atlas[1] **a,** UMAP representations of the concatenated latent embedding space of the fetal chromatin accessibility landscape. Each point represents a combination of tissue, estimated postconceptual age (epa) and cell type. Only observed combinations of cell type and tissue are considered. Points are colored by cell type. The inset shows GO biological processes found enriched in peaks associated with the vascular endothelial cells in biolord's counterfactual predictions over cell type (Methods). GO enrichment was assessed using Genomic Regions Enrichment of Annotations Tool (GREAT)[2]. **b,c,** The correlation matrix between the embedding vectors of the attributes **(b)** epa and **(c)** tissue. **d,** Counterfactual predictions which were obtained over a sample of cells from the placenta tissue, by modifying the cell type attribute reveal cell type specific peaks.

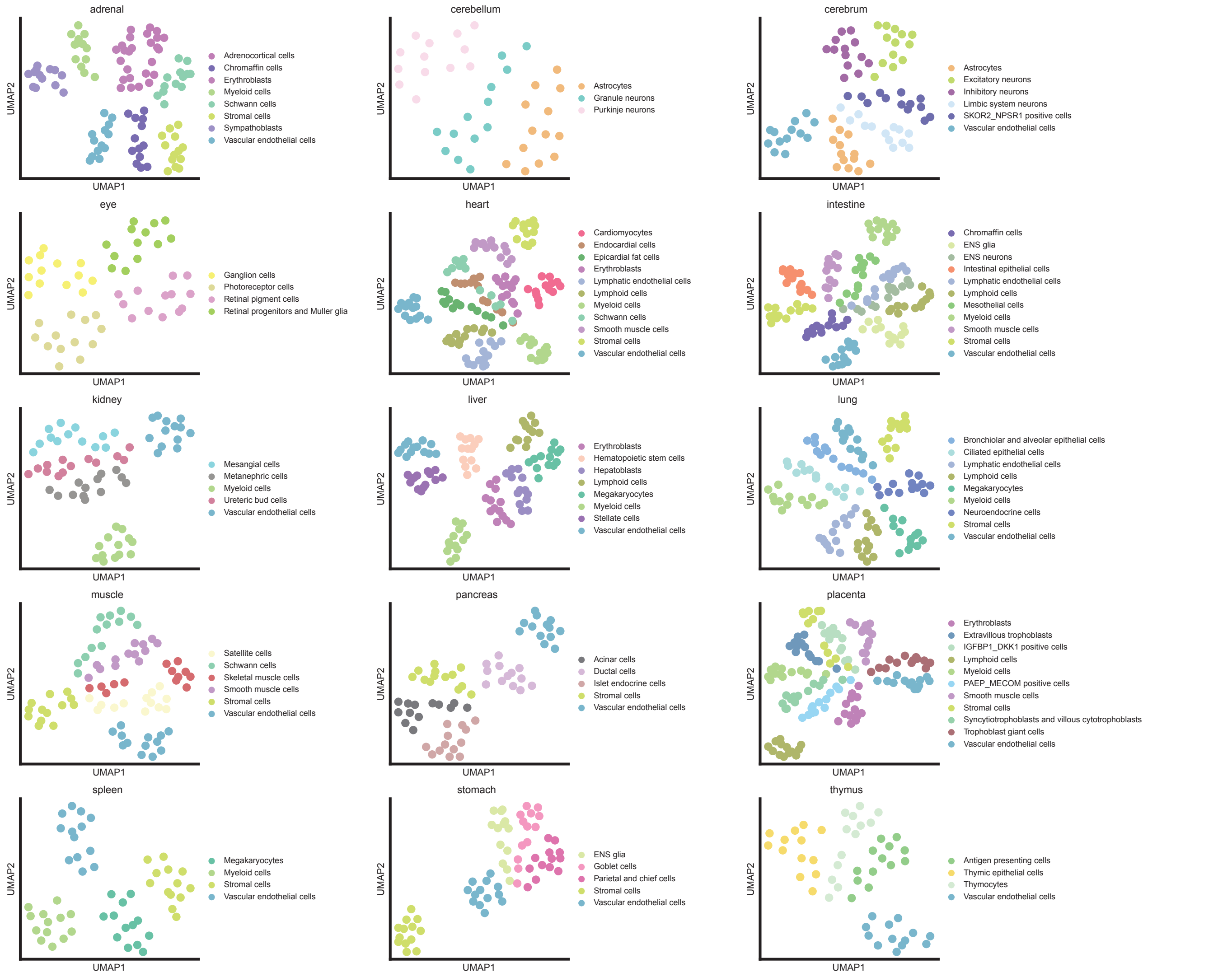

Supplementary Figure 3: The chromatin accessibility landscape latent representation over the 15 different tissues. UMAP visualization of biolord's latent representation for each of the 15 tissues in the data. Each point represents a cell type and epa combination. Colors correspond to cell type.

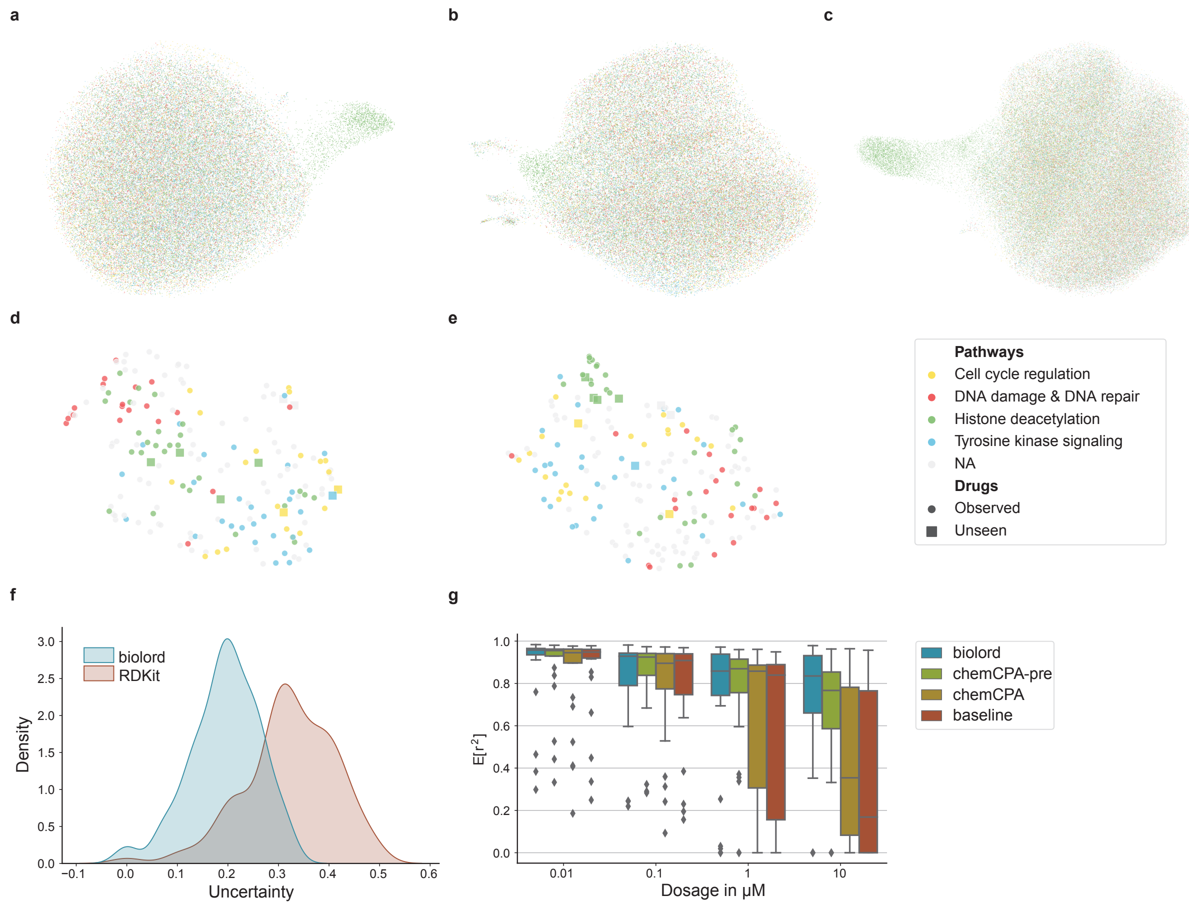

Supplementary Figure 4: Application of biolord for predictions over the sci-Plex 3 dataset[3]. a–c, UMAPs of the original data separated by cell-lines according **(a)** A549 (lung adenocarcinoma), **(b)** K562 (chronic myelogenous leukemia), and **(c)** MCF7 (mammary adenocarcinoma). Cells are colored by pathway associated with the treated-drug. **d,** A UMAP of the chemically informed RDKit features used as input for biolord. **e,** A UMAP of biolord's drug embedding on the highest dosage (10 μM). In both UMAPS, dots, representing drugs, are colored according to known pathways. The shape represents whether the drug is *observed* (circle) or *unseen* (square). **f,** The distribution of the drugs' uncertainty score using biolord latent representations or RDKit features (Methods). **g,** The mean r^2 score, over the nine unseen drugs in each of the three cell-lines and all genes (points are a combination of drug and cell-line resulting in $n = 18$ points). The score is reported for biolord, chemCPA pre-trained model (chemCPA-pre), the standard chemCPA (chemCPA), and the naive baseline (Methods). In the boxplot middle line, median; box boundary, interquartile range (IQR); whiskers, 1.5*IQR; minimum and maximum, not indicated in the box plot; gray dots, points beyond the minimum or maximum whisker.

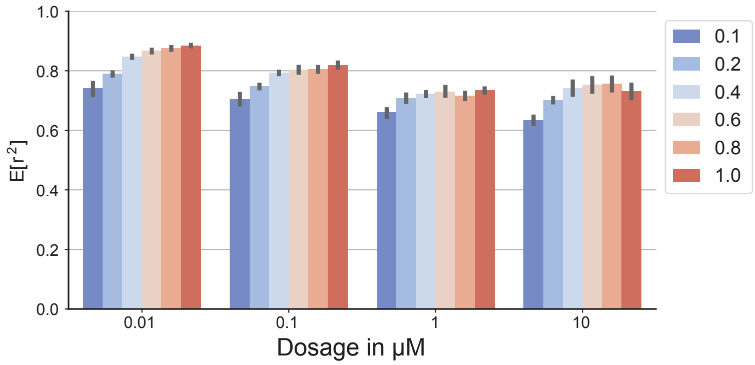

Supplementary Figure 5: Evaluating biolord's robustness. The mean r^2 score of biolord's predictions, over the nine unseen drugs in each of the three cell-lines and all genes (resulting in $n = 18$ data points for evaluation), based on fractions of the sci-Plex 3 dataset[3] (0.1, 0.2, 0.4, 0.6, 0.8, 1). Error bars represent the 95% confidence interval.

Supplementary Note 1

Here we provide further details regarding the architecture, design choices, and training of the biolord and biolord-classify frameworks. In biolord we leverage a combination of a disentanglement framework presented by Gabbay and Hoshen[4] for disentanglement of image attributes, and established probabilistic modeling of single-cell data[5,6].

The biolord framework

As presented in the main text, biolord is a deep learning generative framework, composed of multiple modules which are jointly optimized. As input biolord takes a dataset $\mathcal{D} = [(x_c, y_c)]_{c=1}^{N}$ where for each cell c, $x_c \in \mathbb{R}^M$ are the measured features (e.g. a vector of gene expression counts or peak counts), and y_c is a set of size K representing the known cell attributes. The biolord pipeline consists of two main components, the *decomposed latent space* and the *generative module.* For the biolord-classify, a *classification module* is added. All components are defined and trained jointly with a single objective function.

To obtain the *decomposed latent space* we rely on the concept of latent embedding, as shown to be useful by Gabbay and Hoshen[4]. A latent embedding can be thought of as a simple lookup table that stores the representation (the embedding) of a fixed dictionary. The stored embeddings are optimized directly by the model's objective. This is in contrast to amortized inference, which learns a mapping from the measurements to the embeddings using an encoder. In practice, for the categorical known attributes we use simple Embeddings (using 'torch.nn.Embedding'), and for the unknown attributes we use Regularized Embeddings, embeddings which are regularized by Gaussian noise ($\eta \sim \mathcal{N}(0, \sigma)$) for some fixed variance value σ. Importantly, the latent embeddings are initialized at a random state by the dictionary and hence start from a disentangled state. This may not be the case when considering the initial conditions of an encoder in the amortized setting.

A main challenge in obtaining a meaningful *decomposed latent space* is to limit the information represented in the unknown attribute descriptors. Previous attempts addressed this task in various ways, for example, by setting a bottleneck on the dimension of the embedding or by matching its distribution to a prior normal distribution using KL-divergence (optimizing for both the mean and variance). However, Gabbay and Hoshen[4] observed that using these approaches did not substantially reduce the information represented in the unknown attribute descriptors and suggested the following regularizers instead: (i) adding an activation decay penalty to the loss, limiting the ℓ_2 norm of the unknown embeddings. (ii) Regularizing the embeddings by introducing an additive Gaussian noise of a fixed variance (analogous to optimizing KL-divergence with a fixed variance, thereby optimizing only for the mean). Taken together, the regularization on the ℓ_2 norm limits the value range of each latent embedding, while the Gaussian noise sets a limit on how much information could be encoded given a limit on this value range. Unlike previous approaches which only control the variance of the set of embeddings, this allows some specific codes to have zero variance (Supplementary Table 1).

Given the above, the biolord framework represents cell-specific factors through the unknown attributes embeddings. These capture the distinct state of the cell which is not explained by the known attributes. Amongst possible cell-specific factors are batch effects or alternative biological noise. Hence, while batch or sample labels can be passed as a known attribute, it is neither necessary nor recommended. The known attributes are recommended to account for attributes of biological interest, for which we seek an interpretation of the associated biological features.

To define the generator model, generating cell states from the decomposed latent space, we follow the approach presented by scvi-tools[5]. This approach models the input measurements according to

input type, using Gaussian distribution for log-normalized data (scRNA-seq or scATAC-seq), and a zero-inflated negative binomial (ZINB) for raw gene expression counts or Poisson for raw peak counts. We define the reconstruction and completeness loss terms, using the respective negative log-likelihood (NLL) term. In addition, following the formulation of Gabbay and Hoshen[4], we add a mean squared error (MSE) loss term with respect to the predicted means directly, weighted by the hyperparameter τ. While the MSE term is contained in the NLL of the Gaussian distribution (weighted by the predicted variance), we found that optimizing for it directly improves the model performance (Supplementary Table 1).

Supplementary Table 1: Impact of model hyper-parameters evaluated on the sci-Plex 3 data[3]. We compare the performance over the set of optimal parameters (Supplementary Table 5) to three settings: removing the activation decay penalty ($\lambda = 0$), avoiding embedding regularization ($\sigma = 0$), and removing reconstruction penalty ($\tau = 0$). Mean and variance reported are over 10 random seeds initializations of the model.

	0.01 μM	0.1 μM	1 μM	10 μM
biolord (optim)	0.88 ± 0.00002	0.81 ± 0.0001	0.73 ± 0.0002	0.76 ± 0.0005
biolord ($\lambda = 0$)	0.88 ± 0.00001	0.81 ± 0.0001	0.72 ± 0.0001	0.74 ± 0.006
biolord ($\sigma = 0$)	0.86 ± 0.00001	0.79 ± 0.00002	0.65 ± 0.00008	0.58 ± 0.0005
biolord ($\tau = 0$)	0.42 ± 0.002	0.4 ± 0.002	0.34 ± 0.001	0.25 ± 0.0008

Training a biolord model

To train a biolord model over a given dataset we follow the procedure described by scvi-tools[5]. We first register the data using the anndata registration function. For example, for a dataset with ordered ("age") and categorical ("cell_type" and "tissue") attributes which we want to train over the raw counts, saved in the "counts" layer of the anndata object, we will call,

```
biolord.Biolord.setup_anndata(
    adata,
    ordered_attributes_keys = ["age"],
    categorical_attributes_keys = ["cell_type","tissue"]
    layer = "counts"
)
```

Next, we instantiate a "model" object, which will take as input the registered anndata object along with additional construction parameters, amongst them are the 'module_params' and the 'split_key'. The former allows the user to modify default architecture choices, such as depth and width of the components, and pass the 'gene_likelihood' distribution describing the input data. The 'split_key' provides a division of the data to train, test, and validation sets allowing to both define a set of unseen states to test the model's prediction capabilities and also monitor training to avoid over-fitting over trained data. At last, the user can modify the dimension of the latent vectors of the known attributes ('n_latent_attribute_ordered' and 'n_latent_attribute_discrete') and unknown attributes ('n_latent'). An example call could be,

```
model = biolord.Biolord(
    adata=atac,
    n_latent=128,
    n_latent_attribute_ordered=128,
    n_latent_attribute_discrete=128,
    model_name="my_model",
```

```
    module_params={
        "decoder_width": 512,
        "attribute_nn_depth": 2,
        "gene_likelihood": "nb" # we passed raw counts thus expect NB distribution
    },
    split_key="split",
)
```

To train the model, the user can modify the training procedure, with various evaluation and saving options. The training plan hyper-parameters can be passed using 'plan_kwargs' (following scvi-tools API[5]). Additionally, the user can modify the 'early_stopping' option, relying on a validation set to stop training, the 'batch_size' and 'max_epochs'. An example call to train a model with 'early_stopping' over 100 epochs with batch size of 1024 will be,

```
model.train(
    max_epochs=100,
    batch_size=1024,
    plan_kwargs={
        "step_size_lr": 45,
        "cosine_scheduler": True,
    }
    early_stopping=True,
    early_stopping_patience=20,
)
```

Training a biolord-classify model

Training a biolord-classify model follows a similar procedure to the training pipeline of the biolord model. However, when registering the anndata object we will need to specify the observation category that indicates which labels are missing. For example, assume we have missing "cell_type" annotations for some of the cells, provided in the anndata as "Unknown", we will register the data as follows,

```
biolord.Biolord.setup_anndata(
    adata,
    ordered_attributes_keys = ["age"],
    categorical_attributes_keys = [
        "cell_type",
        "tissue"
    ],
    categorical_attributes_missing = {
        "cell_type": "Unknown",
        "Tissue": None
    }
    layer = "counts"
)
```

Next, when instantiating a model, we will pass "train_classifiers=True" to ensure the classifiers are built and trained,

```
model = biolord.Biolord(
    adata=atac,
    n_latent=128,
    model_name="my_model",
    module_params={
        "decoder_width": 512,
        "attribute_nn_depth": 2,
        "gene_likelihood": "nb" # we passed raw counts thus expect NB distribution
    },
    train_classifiers=True,
    split_key="split",
)
```

Following this, training is performed as presented above for the biolord model.

Applying biolord to chromatin accessibility atlas

Single-cell sequencing assay for transposase-accessible chromatin (scATAC-seq[7]) provides an orthogonal layer of information to the gene expression captured by scRNA-seq. Recent advances in methods for profiling chromatin accessibility allow generation of comprehensive single-cell atlases[1,8]. As we show here, the biolord framework can be applied to such data and find an informative representation with respect to diverse cellular properties (Methods). Specifically, we focus on a human single-cell atlas of fetal chromatin accessibility with samples spanning 89 to 125 days in estimated post-conceptual age (epa) and representing 15 tissues[1]. In the original study, a corresponding gene expression atlas[9] was used to obtain cell type annotations for 54 distinct cell types. In this case, the contributions of tissue, cell type and epa to the final cell state are entangled within this complex landscape, and can be disentangled using biolord (Supplementary Fig. 1a-c, Supplementary Note 2).

Biolord's decomposed latent space reveals the relationships within and across attributes. For each attribute (tissue, cell type and epa), the correlation between the embedding vectors provides a quantification of the relationship between possible states. For example, based on the correlation between the epa values, they are clustered according to their temporal order (89 to 125) and further internal structure between the states is exposed, with a block-wise pattern between temporally-adjacent states (Supplementary Fig. 2b,c). To study the relations between the attributes we consider the lower dimensional representation of the concatenated embeddings, such that each point represents a combination of cell type, tissue and epa (Supplementary Fig. 1d-e, Supplementary Fig. 3; to avoid biologically implausible scenarios we consider only cell type and tissue combinations seen in the original data). In general, the tissue is the dominant attribute, as the majority of the points cluster according to it, and the epa dictates a gradient pattern within each cluster. An exception to this are the vascular endothelial cells which cluster together and internally preserve the tissue association. Namely, biolord identified a strong underlying signature for vascular endothelial cells across all tissues while preserving tissue associated properties (Supplementary Fig. 2a). This is consistent with the results by Domcke et al.[1] in which the vascular endothelial cells appeared as isolated subclusters within each tissue, although they could not be characterized by specific marker genes[1].

To this end, to recover the endothelial specific signature we use biolord to generate counterfactual predictions. Ideally, such counterfactual predictions should capture differences in gene expression induced only by the modified attribute, and thus reveal gene programs related to it (Figure 1c, Methods). We

consider a sample of cells from the placenta tissue and virtually shift the cell type identity of these cells. This revealed cell type specific peaks for the different cell types (Supplementary Fig. 2d, Methods). Applying Genomic Regions Enrichment of Annotations Tool (GREAT)[2,10]. to the peaks associated with the vascular endothelial cells, recovered endothelial associated programs. Amongst them are regulation of endothelial processes as well as angiogenesis and tube morphogenesis, in which endothelial cells are known to play a key role[11,12] (Supplementary Fig. 2d).

Supplementary Note 2

Fetal chromatin accessibility atlas

We use the human cell atlas of fetal chromatin accessibility presented by Domcke et al.[1]. The data was obtained by applying sci-ATAC-seq (a single-cell profiling of chromatin accessibility using combinatorial indexing) to 59 human fetal samples ranging from 89 to 125 days in estimated postconceptual age and representing 15 organs. To analyze the data we downloaded it as an anndata file provided by Cao et al.[9]. The anndata file contains the cell metadata provided by Domcke et al.[1], including: cell_type: the cell type annotation obtained by Domcke et al.[1] using a reference scRNA-seq dataset[9]. Following Domcke et al.[1] we restricted the analysis to the 54 main cell types observed across all tissues. day_of_pregnancy: the estimated postconceptual age of each sample. tissue: the tissue of origin We applied standard pre-processing, normalizing per cell 'scanpy.pp.normalize_per_cell()'), log transforming the peaks ('scanpy.pp.log1p()'), and finding highly variable peaks ('scanpy.pp.highly_variable_genes()'), resulting in 10,616 peaks across 656,074 cells. The biolord model was defined over all cells (656,074 cells). As input, we use the raw peak counts matrix (restricted to the 10,616 highly variable peaks), along with "day_of_pregnancy" (as an ordered attribute), cell_type (as a categorical attribute with 54 cell type annotations), and tissue (as a categorical attribute with 15 tissues categories)

We apply biolord to the sci-ATAC-seq3 dataset curated by Domcke et al.[1]. The relevant anndata file can be downloaded from figshare (atac-tissue-age-celltype). The setting for the biolord model is provided in Supplementary Table 2 and hyperparameter choice in Supplementary Table 3.

Supplementary Table 2: The setting for the biolord model for the fetal chromatin accessibility atlas.

Parameter	Value
ordered_attributes_keys	["day_of_pregnancy"]
categorical_attributes_keys	["cell_type", "tissue"]
train_classifiers	False
batch_size	1024
split_key	"split_random"
max_epochs	100
early_stopping	False

Vascular endothelial cells signature analysis

We obtained counterfactual predictions by considering cells from a single sample, taken from the placenta tissue, modified their cell type attribute (considering all 11 cell types identified in the placenta tissue) and obtained a predicted peak counts matrix. Using 'scanpy.tl.rank_genes_groups()' we identified peaks associated with each cell type (Supplementary Fig. 2). We used Genomic Regions Enrichment of Annotations Tool (GREAT)[2,10] to identify biological programs enriched in the vascular endothelial cells. We considered as input to GREAT the top 500 peaks associated with the vascular endothelial cells and all highly variable peaks as background.

Supplementary Table 3: Parameters of the biolord model used for the fetal chromatin accessibility atlas.

Parameter	Value
n_latent	128
n_latent_attribute_ordered	16
n_latent_attribute_categorical	16
decoder_width	512
decoder_depth	6
decoder_lr	1×10^{-4}
decoder_wd	1×10^{-4}
attribute_nn_lr	1×10^{-4}
attribute_nn_wd	4×10^{-8}
unknown_attribute_noise_param	1×10^{0}
reconstruction_penalty	1×10^{1}
unknown_attribute_penalty	1×10^{9}
step_size_lr	45
use_batch_norm	False
use_layer_norm	False
cosine_scheduler	True
attribute_dropout_rate	0.1
scheduler_final_lr	1×10^{-5}
gene_likelihood	"poisson"

Supplementary Note 3

Predicting cellular responses to perturbations

Advancements in experimental methods made it possible to generate extensive drug and genetic perturbation datasets on a large scale. However, experimentally exploring the space of all possible combinatorial perturbations is in general not feasible. Therefore, there is a need for computational tools that can limit this space by predicting the effects of unseen perturbations based on existing data. Indeed, this subject is of great interest and recently various computational methods dedicated for this task have been suggested. However, many of the suggested methods cannot generalize to completely unseen states. For example scGEN[13] and CPA[14] by Lotfollahi et al., deep learning frameworks which are pioneer works in the field, can only make predictions over unobserved combinations of drugs and cell lines, with the requirement that the individual components have been seen during training. That is each drug, cell line, and dose must be present in the training data independently, and then predictions over unseen combinations are made. Another line of work, CellOT[15] and CondOT[16] by Bunne et al., address this task using tools from Optimal Transport theory. A transport map provides a coupling between two distributions representing perturbed or unperturbed cells. Using the coupling predictions can be made over unseen unperturbed cells. In this setting the focus is on learning patient-specific response, that is, making predictions with respect to held out cells and not treatments.

Amongst the methods that allow for counterfactual predictions over unseen states are the following deep generative models: chemCPA[17] by Hetzel et al., for drug perturbations, GEARS[18] by Roohani et al., for genetic perturbations, and PerturbNet[19] by Yu et al., which addresses both. As we elaborate below, all of these methods use additional prior knowledge concerning the task, introduce pre-training steps to incorporate it and tailor the architecture for this setting.

chemCPA[17] is an encoder-decoder architecture which incorporates prior knowledge about the compounds' structure to allow for generalization to unseen drugs. The framework relies on the CPA[14] construction, namely, given a single-cell drug perturbation dataset, a set of encoder neural network are used to encode the cells' gene expression, perturbation, and possibly additional covariates (e.g. cell-type or drug dosage). These define a compositional latent space which is used as input for a decoder. The encoder-decoder setting is adversarially trained using adversarial classifiers. chemCPA introduces a perturbation network for the perturbations encoding. This network takes as input a molecular representation of the drug, instead of a categorical label, and the dosage. As the encoding is based on molecular representation the model can generalize to unseen drugs through feature resemblance. Along with a drug embedding a dosage scalar is trained over chemical representation and the dosage and outputs a scaled dosage scalar. The perturbation network output is defined as a multiplication of the scaled dosage and drug embedding. To further utilize existing prior knowledge, the authors suggest pre-training this network, independently of model training, over bulk RNA high throughput screens (HTS) to improve generalization. This is the setting referred to as chemCPA-pre in the text. In this scenario, the chemCPA model uses a perturbation network which was trained over a large bulk RNA HTS, and hence effectively *seen* a larger set of perturbations then those present in the single-cell data.

The perturbation network defines a major difference between chemCPA and biolord. While biolord uses a chemical representation to allow for generalization, there is no tailored procedure to model the dosage. Further, the pre-training procedure exposes the model to data which is never seen by the biolord model. In addition, chemCPA uses adversarial training to induce disentanglement whereas in biolord we perform latent optimization (see Supplementary Note 1).

GEARS[18] is a deep learning framework based on Graph Neural Networks (GNNs) for multi-gene perturbation predictions. GEARS represents each gene and each perturbation using its own embedding

vector by defining two GNN-based encoders. The first encodes observed gene expression to a gene embedding, namely providing a gene co-expression graph. Next, a Gene Ontology graph is used to define the GNN for the perturbation encoding, therefor leveraging prior knowledge and allowing for generalization to unseen perturbations. To model multi-gene perturbations, capturing non-linear effects, given a set of perturbations an MLP is defined over the sum of the independent embeddings. At last, For each gene, a gene-specific linear layer is learnt to couple the embedding of the observed perturbations with the gene embedding, resulting in a "perturbed" gene embedding. In addition a cross-gene embedding is learnt, aimed to capture secondary effects.

In biolord, we utilize the Gene Ontology graph, using the adjacency matrix as input features representing the gene perturbations as an ordered attribute. This allows us to generalize to unseen gene perturbations within the biolord framework. Preserving the generality of biolord we do not offer a specific module to model multi-gene perturbations, and our model provides predictions for single gene perturbations. However, as we show in the main text, over the existing settings, as in the two gene predictions task, using a summation of our single gene perturbations, biolord predictions outperform available baselines (Extended Data Fig. 2).

PerturbNet[19] is a deep generative model for predicting the distribution of cell states induced by unseen drugs or genetic perturbations. The PerturbNet model consists of three neural networks, trained end-to-end independently: (1) a perturbation representation network, (2) a cellular representation network, and a (3) network mapping from perturbations to cell states. First, each representation network is trained, over large datasets of its domain, to learn a meaningful latent representation. These networks also train a decoder, mapping from the latent state to the perturbation or expression, respectively. Next, the mapping network is trained to map between the latent representations. Using a high-throughput perturbation data which contains both domains, the latent representations are obtained by the pre-trained (1)+(2) and the mapping network provides the matching between them (i.e. the perturbation and cell states). To obtain perturbation or expression predictions, the mapped states are inputted to the dedicated decoder.

In contrast to PerturbNet, in biolord we considered a joint end-to-end training of the model components, looking to find a decomposed latent space which maximizes the prediction accuracy. Following this, biolord provides counterfactual predictions directly on the level of measurement space, and does not offer a mapping to cell latent space. Given the above, while PerturbNet allows to accurately predict cell states and further design perturbations to achieve target cell state distributions, it could not accurately predict gene expression response over unseen drugs (see sci-Plex 3).

sci-Plex 3

biolord.

For all biolord models of the sci-Plex 3 dataset[3] we use the same anndata file available on figshare (sci-plex3) and the biolord model settings as listed in Supplementary Table 4. We used Weights & Biases[20] for experiment tracking and hyper-parameter tuning. To cover the large space of possible configuration space we tuned subsets of the parameters in consecutive iterations (Supplementary Table 5). We started with tuning the more dominant parameters. For example, "gene_likelihood" or architecture parameters such as "n_latent", and "{}_depth" or "{}_width" parameters. We next tuned the loss parameters (e.g., penalties strength) and the dropout rate. Finally, we tuned the finer optimization parameters such as the learning rates and regularization parameters. We randomized over valid values for the inspected parameters at each iteration and kept the ones that consistently outperformed. The range of values scanned for each parameter and the best configurations are given in Supplementary Table 5.

Supplementary Table 4: General settings for a biolord model on the sci-Plex 3 data[3].

Parameter	Value
ordered_attributes_keys	["rdkit2d_dose"]
discrete_attributes_keys	["cell_type"]
train_classifiers	False
batch_size	512
split_key	"split_ood"
max_epochs	500
early_stopping	True
gene_likelihood	"normal"
unknown_attributes	True
decoder_activation	True
attribute_nn_activation	True

Supplementary Table 5: Parameters range for the biolord sweep on the sci-Plex 3 data[3] and optimal configuration used.

Parameter	Values	Best config
n_latent	[16,32,64,128,256]	256
n_latent_attribute_ordered	[128,256,512]	256
n_latent_attribute_discrete	[2,3,4,6,8]	3
latent_lr	$[1 \times 10^{-2}, 1 \times 10^{-3}, 1 \times 10^{-4}]$	1×10^{-4}
latent_wd	$[1 \times 10^{-2}, 1 \times 10^{-3}, 1 \times 10^{-4}]$	1×10^{-4}
decoder_width	[32,64,128,256,512,1024,2048,4096]	4096
decoder_depth	[1,2,3,4,6,8]	4
decoder_lr	$[1 \times 10^{-2}, 1 \times 10^{-3}, 1 \times 10^{-4}]$	1×10^{-4}
decoder_wd	$[1 \times 10^{-2}, 1 \times 10^{-3}, 1 \times 10^{-4}]$	1×10^{-4}
attribute_nn_width	[32,64,128,256,512,1024,2048,4096]	2048
attribute_nn_depth	[1,2,3,4,6,8]	2
attribute_nn_lr	$[1 \times 10^{-2}, 1 \times 10^{-3}, 1 \times 10^{-4}]$	1×10^{-2}
attribute_nn_wd	$[1 \times 10^{-8}, 4 \times 10^{-8}, 1 \times 10^{-7}]$	4×10^{-8}
unknown_attribute_noise_param	$[0.1, 0.5, 1, 2, 5, 10, 20]$	20
reconstruction_penalty	$[1 \times 10^{-2}, 1 \times 10^{-1}, 1 \times 10^{0}, 1, 1 \times 10^{2}, 5 \times 10^{3}, 1 \times 10^{4}]$	1×10^{4}
unknown_attribute_penalty	$[0.1, 0.5, 1, 2, 5, 10, 20, 50, 100, 200]$	0.5
step_size_lr	$[45, 90, 180]$	45
use_batch_norm	[True, False]	False
use_layer_norm	[True, False]	False
cosine_scheduler	[True, False]	True
attribute_dropout_rate	[0.05,0.1,0.25,0.5,0.75]	0.1
scheduler_final_lr	$[1 \times 10^{-3}, 1 \times 10^{-4}, 1 \times 10^{-5}, 1 \times 10^{-6},]$	1×10^{-5}

Benchmarks

chemCPA. For the non-pre-trained version, we follow the parameters supplied in 'finetuning_num_genes.json' ('_id = 1007')[17]. The parameters are presented in Supplementary Table 6.

chemCPA-pre. For the pre-trained version, we followed 'finetuning_num_genes.json'[17] ('_id = 789'). As advised by the authors we tune the adversarial parameters, as detailed in Supplementary Table 7.

Supplementary Table 6: Hyperparameters used for reported chemCPA results on the sci-Plex 3 data[3].

Parameter	Value
num epochs	200
patience	50
dim	32
dropout	2.624×10^{-1}
autoencoder_width	256
autoencoder_depth	4
autoencoder_lr	1.575×10^{-3}
autoencoder_wd	6.251×10^{-7}
adversary_width	128
adversary_depth	3
adversary_lr	8.060×10^{-4}
adversary_wd	4.000×10^{-6}
reg_adversary	9.101×10^{0}
reg_adversary_cov	1.068×10^{1}
penalty_adversary	4.550×10^{-1}
batch_size	32
dosers_width	64
dosers_depth	3
dosers_lr	1.575×10^{-3}
dosers_wd	6.251×10^{-7}
embedding_encoder_width	128
embedding_encoder_depth	4
append_ae_layer	True
enable_cpa_mode	False
reg_multi_task	0

Supplementary Table 7: Parameters range for the chemCPA-pre sweep on the sci-Plex 3 data[3] and optimal configuration used.

Parameter	Values	Best config
num epochs	-	200
patience	-	50
dim	-	32
dropout	-	2.624×10^{-1}
autoencoder_width	-	256
autoencoder_depth	-	4
autoencoder_lr	-	2.051×10^{-4}
autoencoder_wd	-	2.940×10^{-8}
reg_adversary_cov	-	4.176
batch_size	-	32
dosers_width	-	64
dosers_depth	-	3
dosers_lr	-	2.051×10^{-4}
dosers_wd	-	2.940×10^{-8}
embedding_encoder_width	-	128
embedding_encoder_depth	-	4
append_ae_layer	-	True
enable_cpa_mode	-	False
reg_multi_task	-	0
adversary_width	$[64, 128, 256]$	256
adversary_depth	$[2, 3, 4]$	3
adversary_lr	$(5 \times 10^{-5}, 1 \times 10^{-2})$	1.143×10^{-4}
adversary_wd	$(1 \times 10^{-8}, 1 \times 10^{-2})$	4×10^{-6}
adversary_steps	$[2, 3]$	2
reg_adversary	$(5, 100)$	1.778
penalty_adversary	$(0.5, 5)$	8.89×10^{-2}

PerturbNet

A PerturbNet model requires three networks. To train a model for the sci-Plex3 task we follow the flow provided by the authors in perturbnet_sciplex_example_notebook.ipynb. We trained the independent networks following examples in /PerturbNet/perturbnet/perturb, as we detail below:

1. *Perturbation Representation Network*: We used the pre-trained ChemicalVAE provided by the authors, as used in the sci-Plex3 example and provided here.
2. *Cellular Representation Network*: We train an scVI model following the setting in main_scvi_overall.py. As input we use the same AnnData file we used in all sci-Plex3 evaluations, (sciplex3), removing the 9 unseen drugs from training.
3. *Mapping network (cINN)*: We followed the procedure presented in chemvae_covariates_scvi_flow_train.py, training a cINN model with additional covariates using the latent representation of the pretrained networks, (1) and (2).

To obtain counterfactual predictions using the PerturbNet model we considered two approaches:

1. *Translation*: We followed the definition of Yu et al.[19], suggesting the counterfactual prediction setting with respect to the translation task, which aims to find an alternative perturbation mapping a cell to a new state. Within this task the function, "trans_data(latent=latent_ref, condition=cond_ref, condition_new=cond_unseen, ..)" is used to obtain a prediction of the expression (and cell state) of a reference cell (defined by "latent_ref" and "cond_ref") if it were to be perturbed by "cond_unseen". This setting requires the reference cell to have a defined condition ("ctrl" state is not modeled) by PertrubNet). Hence, for each "cond_unseen", a combination of unseen drug, cell line and dosage, we fix the cell line and dosage, and iterate over all seen drugs. We obtain the expression prediction using "trans_data()" and evaluate the performance using the r^2 score (as done for other frameworks). For each unseen condition we report the maximal score obtained (maximal amongst the comparisons to all seen drugs).
2. *Control*: In Yu et al.[19] the expression prediction task is presented over drugs which are unseen during training using the function "recon_data(latent=latent_unseen, condition_new=cond_unseen, ..)". However, to obtain "latent_unseen" the Cellular representation network (the scVI model) is provided with the expression of the "unseen" cells. To use this procedure for "counterfactual" predictions, we utilize the fact that the scVI model is oblivious to the perturbations, and obtain a cell state representation of control cells ("latent_control"). We use these instead of "latent_unseen" to obtain predictions using `recon_data()`, again matching the cell line of control and evaluated cells. We report the mean r^2 score between predictions and observed expression.

Results are presented in Supplementary Table 8. However, as mentioned above, the PerturbNet model is optimized to map cell state representation, and not expression prediction.

Supplementary Table 8: PerturbNet and biolord model mean r^2 prediction score on the sci-Plex 3 data over the nine unseen drugs and all genes. Results are reported for all four dosage values.[3]

	0.01 μM	0.1 μM	1 μM	10 μM
PerturbNet (translation)	0.19	0.17	0.15	0.1
PerturbNet (control)	0.61	0.51	0.4	0.22
biolord	0.88	0.8	0.72	0.74

Perturb-seq (one-gene)

biolord. For the Perturb-seq (one-gene) dataset by Adamson et al.[21] we use the anndata files available on figshare (perturbseq_adamson and perturbseq_adamson_single). We train the biolord model using the settings as listed in Supplementary Table 9 over five different splits. Weights & Biases[20] was used for experiment tracking and hyper-parameter tuning. To cover the large space of possible configuration space we tuned subsets of the parameters in consecutive iterations (Supplementary Table 10). We jointly optimized the set of parameters to find the final reported configuration. The range of values scanned for each parameter and the best configurations are given in Supplementary Table 10. We used the same set of parameters to evaluate over all splits.

Supplementary Table 9: General settings for a biolord model on the Perturb-seq (one-gene) dataset[21].

Parameter	Value
ordered_attributes_keys	["perturbation_neighbors"]
batch_size	32
n_latent	32
split_key	"split1" or "split2" or "split3" or "split4" or "split5"
early_stopping	True
gene_likelihood	"normal"
scheduler_final_lr	1×10^{-5}
step_size_lr	45
unknown_attributes	False
train_classifiers	False
use_batch_norm	False
use_layer_norm	False
cosine_scheduler	True

Supplementary Table 10: Parameters range for the biolord sweep on the Perturb-seq (one-gene) dataset[21] and optimal configuration used.

Parameter	Values	Best config
n_latent_attribute_ordered	[32, 64, 128, 256, 512]	512
latent_lr	$[1 \times 10^{-1}, 1 \times 10^{-2}, 1 \times 10^{-3}, 1 \times 10^{-4}, 1 \times 10^{-5}]$	1×10^{-4}
latent_wd	$[1 \times 10^{-2}, 1 \times 10^{-3}, 1 \times 10^{-4}, 1 \times 10^{-5}]$	1×10^{-3}
decoder_width	[32,64,128,256, 512]	64
decoder_depth	[1,2,3,4,6,8]	1
decoder_lr	$[1 \times 10^{-2}, 1 \times 10^{-3}, 1 \times 10^{-4}]$	1×10^{-3}
decoder_wd	$[1 \times 10^{-2}, 1 \times 10^{-3}, 1 \times 10^{-4}]$	1×10^{-2}
attribute_nn_width	[32,64,128,256,512]	64
attribute_nn_depth	[1,2,3,4,6,8]	6
attribute_nn_lr	$[1 \times 10^{-2}, 1 \times 10^{-3}, 1 \times 10^{-4}]$	1×10^{-3}
attribute_nn_wd	$[4 \times 10^{-9}, 4 \times 10^{-8}, 4 \times 10^{-7}]$	4×10^{-8}
reconstruction_penalty	$[1 \times 10^{-1}, 1 \times 10^{0}, 1, 1 \times 10^{2}, 1 \times 10^{3}, 1 \times 10^{4}, 1 \times 10^{5}]$	1×10^{3}
decoder_activation	[True, False]	False
attribute_nn_activation	[True, False]]	False
attribute_dropout_rate	[0.0, 0.1,0.25,0.5,0.75]	0.1
max_epochs	[200, 500, 1000]	1000
early_stopping_patience	[10, 50, 100, 200]	10

Perturb-seq (two-gene)

biolord. For the Perturb-seq (two-gene) dataset by Norman et al.[22] we use the anndata files available on figshare (perturbseq_norman and perturbseq_norman_single). We train the biolord model using the settings as listed in Supplementary Table 11 over five different splits. We train the model over the one-gene perturbations in the dataset and use it to predict the two-gene perturbations by modeling $\Delta(g_1 + g_2) = \Delta g_1 + \Delta g_2$. As in previous datasets, Weights & Biases[20] was used for experiment tracking and hyper-parameter tuning. The range of values scanned for each parameter and the best configurations are given in Supplementary Table 12. We used the same set of parameters to evaluate over all splits.

Supplementary Table 11: General settings for a biolord model on the Perturb-seq (two-gene) dataset[22].

Parameter	Value
ordered_attributes_keys	["perturbation_neighbors1"]
batch_size	32
n_latent	32
split_key	"split1" or "split2" or "split3" or "split4" or "split5"
early_stopping	True
gene_likelihood	"normal"
scheduler_final_lr	1×10^{-5}
step_size_lr	45
unknown_attributes	False
train_classifiers	False
use_batch_norm	False
use_layer_norm	False
cosine_scheduler	True

Supplementary Table 12: Parameters range for the biolord sweep on the Perturb-seq (two-gene) dataset[22] and optimal configuration used.

Parameter	Values	Best config
n_latent_attribute_ordered	[32, 64, 128, 256, 512]	32
latent_lr	$[1 \times 10^{-1}, 1 \times 10^{-2}, 1 \times 10^{-3}, 1 \times 10^{-1}, 1 \times 10^{-5}]$	1×10^{-1}
latent_wd	$[1 \times 10^{-2}, 1 \times 10^{-3}, 1 \times 10^{-4}, 1 \times 10^{-5}]$	1×10^{-5}
decoder_width	[32,64,128,256, 512]	32
decoder_depth	[1,2,3,4,6,8]	2
decoder_lr	$[1 \times 10^{-2}, 1 \times 10^{-3}, 1 \times 10^{-4}]$	1×10^{-2}
decoder_wd	$[1 \times 10^{-2}, 1 \times 10^{-3}, 1 \times 10^{-4}]$	1×10^{-2}
attribute_nn_width	[32,64,128,256,512]	64
attribute_nn_depth	[1,2,3,4,6,8]	2
attribute_nn_lr	$[1 \times 10^{-2}, 1 \times 10^{-3}, 1 \times 10^{-4}]$	1×10^{-3}
attribute_nn_wd	$[4 \times 10^{-9}, 4 \times 10^{-8}, 4 \times 10^{-7}]$	4×10^{-8}
reconstruction_penalty	$[1 \times 10^{-1}, 1 \times 10^{0}, 1, 1 \times 10^{2}, 1 \times 10^{3}, 1 \times 10^{4}, 1 \times 10^{5}]$	1×10^{4}
decoder_activation	[True, False]	True
attribute_nn_activation	[True, False]	True
attribute_dropout_rate	[0.0, 0.1,0.25,0.5,0.75]	0.0
max_epochs	[200, 500, 1000]	200
early_stopping_patience	[10, 50, 100, 200]	200

Supplementary Note 4

Spatio-temporal single-cell atlas of the Plasmodium liver stage

As described in the main text we define two biolord settings for the analysis of the spatio-temporal single-cell atlas of the Plasmodium liver stage[23]. Below we provide the model details for each case.

Infected state analysis using counterfactual predictions

The relevant anndata file can be downloaded from figshare (spatio-temporal-infection_infected). The setting for the biolord model is provided in Supplementary Table 13 and hyperparameter choice in Supplementary Table 14. Of note, parameters relating to ordered attributes are missing as we do not have ordered attributes.

Supplementary Table 13: The setting for the biolord model for the infected state analysis.

Parameter	Value
discrete_attributes_keys	["time_int", "status_control", "zone"]
train_classifiers	False
batch_size	512
split_key	"split_random"
max_epochs	500
early_stopping	True
early_stopping_patience	20

Supplementary Table 14: Parameters of the biolord model used for the infected state analysis.

Parameter	Value
n_latent	32
n_latent_attribute_categorical	4
decoder_width	1024
decoder_depth	4
decoder_lr	1×10^{-4}
decoder_wd	1×10^{-4}
attribute_nn_lr	1×10^{-4}
attribute_nn_wd	1×10^{-4}
unknown_attribute_noise_param	1×10^{-1}
reconstruction_penalty	1×10^{2}
unknown_attribute_penalty	1×10^{1}
step_size_lr	45
use_batch_norm	False
use_layer_norm	False
cosine_scheduler	True
attribute_dropout_rate	0.1
scheduler_final_lr	1×10^{-5}
gene_likelihood	"normal"

Abortive state classification

The relevant anndata file can be downloaded from figshare (spatio-temporal-infection_abortive), named 'spatio-temporal-infection_abortive'. The setting for the biolord model is provided in Supplementary Table 15 and hyperparameter choice in Supplementary Table 16.

Supplementary Table 15: The setting for the biolord model for the abortive state analysis.

Parameter	Value
ordered_attributes_keys	["stress_score"]
categorical_attributes_keys	["time_int", "abortive_state", "zone"]
categorical_attributes_missing	{"time_int": None, "abortive_state": "Unknown", "zone": None}
train_classifiers	True
batch_size	256
split_key	"split_random"
max_epochs	500
early_stopping	True
early_stopping_patience	20
unknown_attributes	True
decoder_activation	True
attribute_nn_activation	True

Supplementary Table 16: Parameters of the biolord model used for the infected state analysis.

Parameter	Value
n_latent	32
n_latent_attribute_categorical	4
n_latent_attribute_ordered	16
decoder_width	512
decoder_depth	4
decoder_lr	1×10^{-4}
decoder_wd	1×10^{-4}
latent_lr	1×10^{-4}
latent_wd	1×10^{-4}
attribute_nn_width	512
attribute_nn_depth	4
attribute_nn_lr	1×10^{-2}
attribute_nn_wd	4×10^{-8}
unknown_attribute_noise_param	1×10^{-1}
reconstruction_penalty	1×10^{2}
unknown_attribute_penalty	1×10^{1}
step_size_lr	90
use_batch_norm	False
use_layer_norm	False
cosine_scheduler	True
attribute_dropout_rate	0.05
scheduler_final_lr	1×10^{-5}
gene_likelihood	"normal"
loss_regression	"normal"
classification_penalty	0
classifier_dropout_rate	1×10^{-1}
classifier_penalty	1×10^{1}
classify_all	False
unknown_attributes	True
decoder_activation	True
attribute_nn_activation	True

References

[1] Silvia Domcke, et al. A human cell atlas of fetal chromatin accessibility. *Science*, 370(6518):eaba7612, 2020.

[2] Cory Y McLean, et al. Great improves functional interpretation of cis-regulatory regions. *Nature biotechnology*, 28(5):495–501, 2010.

[3] Sanjay R Srivatsan, et al. Massively multiplex chemical transcriptomics at single-cell resolution. *Science*, 367(6473):45–51, 2020.

[4] Aviv Gabbay and Yedid Hoshen. Demystifying inter-class disentanglement. In *International Conference on Learning Representations*, 2020.

[5] Adam Gayoso, et al. A python library for probabilistic analysis of single-cell omics data. *Nature biotechnology*, 40(2):163–166, 2022.

[6] Laura D Martens, et al. Modeling fragment counts improves single-cell atac-seq analysis. *Nature Methods*, pages 1–4, 2023.

[7] Jason D Buenrostro, et al. Single-cell chromatin accessibility reveals principles of regulatory variation. *Nature*, 523(7561):486–490, 2015.

[8] Kai Zhang, et al. A single-cell atlas of chromatin accessibility in the human genome. *Cell*, 184(24):5985–6001, 2021.

[9] Junyue Cao, et al. A human cell atlas of fetal gene expression. *Science*, 370(6518):eaba7721, 2020.

[10] Yosuke Tanigawa, et al. WhichTF is functionally important in your open chromatin data? *PLOS Computational Biology*, 18(8):e1010378, 2022.

[11] B Ren. Endothelial cells: a key player in angiogenesis and lymphangiogenesis. *MOJ Cell Sci Report*, 2(1): 00015, 2015.

[12] Saravana K Ramasamy, et al. Regulation of tissue morphogenesis by endothelial cell-derived signals. *Trends in cell biology*, 25(3):148–157, 2015.

[13] Mohammad Lotfollahi, et al. scgen predicts single-cell perturbation responses. *Nature methods*, 16(8): 715–721, 2019.

[14] Mohammad Lotfollahi, et al. Predicting cellular responses to complex perturbations in high-throughput screens. *Molecular Systems Biology*, page e11517, 2023.

[15] Charlotte Bunne, et al. Learning single-cell perturbation responses using neural optimal transport. *Nature methods*, pages 1–10, 2023.

[16] Charlotte Bunne, et al. Supervised training of conditional monge maps. In *Advances in Neural Information Processing Systems*, 2022.

[17] Leon Hetzel, et al. Predicting cellular responses to novel drug perturbations at a single-cell resolution. In *Advances in Neural Information Processing Systems*, 2022.

[18] Yusuf Roohani, et al. Predicting transcriptional outcomes of novel multigene perturbations with gears. *Nature Biotechnology*, pages 1–9, 2023.

[19] Hengshi Yu and Joshua D Welch. Perturbnet predicts single-cell responses to unseen chemical and genetic perturbations. *bioRxiv*, pages 2022–07, 2022.

[20] Lukas Biewald. Experiment tracking with weights and biases, 2020. URL https://www.wandb.com/. Software available from wandb.com.

[21] Britt Adamson, et al. A multiplexed single-cell crispr screening platform enables systematic dissection of the unfolded protein response. *Cell*, 167(7):1867–1882, 2016.

[22] Thomas M Norman, et al. Exploring genetic interaction manifolds constructed from rich single-cell phenotypes. *Science*, 365(6455):786–793, 2019.

[23] Amichay Afriat, et al. A spatiotemporally resolved single-cell atlas of the plasmodium liver stage. *Nature*, pages 1–7, 2022.
