# Supplementary material for: Disentanglement of single-cell data with biolord
Source: Nat Biotechnol. 2024 Jan 15;42(11):1678–83. doi: 10.1038/s41587-023-02079-x (PMC11554562; doi:10.1038/s41587-023-02079-x)
Supplement: Supplementary file 2 — Reporting Summary [file 41587_2023_2079_MOESM2_ESM.pdf]

nature portfolio

Corresponding author(s): Dr. Mor Nitzan

Last updated by author(s): Nov 17, 2023

Reporting Summary

Nature Portfolio wishes to improve the reproducibility of the work that we publish. This form provides structure for consistency and transparency in reporting. For further information on Nature Portfolio policies, see our Editorial Policies and the Editorial Policy Checklist.

Statistics

For all statistical analyses, confirm that the following items are present in the figure legend, table legend, main text, or Methods section.

n/a	Confirmed	
☐	☒	The exact sample size (*n*) for each experimental group/condition, given as a discrete number and unit of measurement
☒	☐	A statement on whether measurements were taken from distinct samples or whether the same sample was measured repeatedly
☐	☒	The statistical test(s) used AND whether they are one- or two-sided<br>*Only common tests should be described solely by name; describe more complex techniques in the Methods section.*
☐	☒	A description of all covariates tested
☐	☒	A description of any assumptions or corrections, such as tests of normality and adjustment for multiple comparisons
☐	☒	A full description of the statistical parameters including central tendency (e.g. means) or other basic estimates (e.g. regression coefficient) AND variation (e.g. standard deviation) or associated estimates of uncertainty (e.g. confidence intervals)
☐	☒	For null hypothesis testing, the test statistic (e.g. *F*, *t*, *r*) with confidence intervals, effect sizes, degrees of freedom and *P* value noted<br>*Give P values as exact values whenever suitable.*
☒	☐	For Bayesian analysis, information on the choice of priors and Markov chain Monte Carlo settings
☒	☐	For hierarchical and complex designs, identification of the appropriate level for tests and full reporting of outcomes
☐	☒	Estimates of effect sizes (e.g. Cohen's *d*, Pearson's *r*), indicating how they were calculated

Our web collection on statistics for biologists contains articles on many of the points above.

Software and code

Policy information about availability of computer code

Data collection	No software was used for data collection.
Data analysis	We used biolord v0.0.2.<br>We share our software in an open-source package on github (https://biolord.readthedocs.io/en/latest/) as well as jupyter notebooks to reproduce our figures and analysis (https://https://github.com/nitzanlab/biolord_reproducibility).<br>Python packages: cell-gears v0.0.2, chemprop v1.5.2, muon v0.1.3, numpy v1.22.4, scanpy v1.9.1, scipy v1.10.0, scvi-tools v0.20.3, statsmodels v0.13.5.<br>Python cloned repositories: https://github.com/welch-lab/PerturbNet (commit d719212aab1eb7cc56d7413e611cdb67987f1aec), https://github.com/theislab/chemCPA (commit a4a4ded0c3b949c64ff1ea51033be1b7c301c36b).<br>R packages: clusterProfiler v4.7.1.001, msigdbr v7.5.1, org.Mm.eg.db v3.16.0.

For manuscripts utilizing custom algorithms or software that are central to the research but not yet described in published literature, software must be made available to editors and reviewers. We strongly encourage code deposition in a community repository (e.g. GitHub). See the Nature Portfolio guidelines for submitting code & software for further information.

March 2021

Data

Policy information about availability of data

All manuscripts must include a data availability statement. This statement should provide the following information, where applicable:

- Accession codes, unique identifiers, or web links for publicly available datasets
- A description of any restrictions on data availability
- For clinical datasets or third party data, please ensure that the statement adheres to our policy

The datasets analyzed in the current study are available at:
1. sci-Plex3: https://f003.backblazeb2.com/file/chemCPA-datasets/sciplex_complete_middle_subset.h5ad, a pre-processed file provided by Hetzel et al.
2. Perturb-seq (1-gene): https://dataverse.harvard.edu/api/access/datafile/6154020, pre-processed data and additional files provided by Roohani et al.
3. Perturb-seq (2-gene): https://dataverse.harvard.edu/api/access/datafile/6894431, pre-processed data and additional files provided by Roohani et al.
4. Fetal chromatin accessibility atlas: http://download.gao-lab.org/GLUE/dataset/Domcke-2020.h5ad, a pre-processed file provided by Cao et al.
5. Spatio-temporal single-cell atlas of the Plasmodium liver stage: publicly available at GSE181725 (https://www.ncbi.nlm.nih.gov/geo/query/acc.cgi?acc=GSE181725) or as processed Seurat object at https://zenodo.org/record/7081863.

Human research participants

Policy information about studies involving human research participants and Sex and Gender in Research.

Reporting on sex and gender	not applicable
Population characteristics	not applicable
Recruitment	not applicable
Ethics oversight	not applicable

Note that full information on the approval of the study protocol must also be provided in the manuscript.

Field-specific reporting

Please select the one below that is the best fit for your research. If you are not sure, read the appropriate sections before making your selection.

☒ Life sciences ☐ Behavioural & social sciences ☐ Ecological, evolutionary & environmental sciences

For a reference copy of the document with all sections, see nature.com/documents/nr-reporting-summary-flat.pdf

Life sciences study design

All studies must disclose on these points even when the disclosure is negative.

Sample size	We used five different datasets in this study to showcase the range of possible applications of the biolord method. These datasets provide diversity in terms of the biological system, including mammalian, human and cell-lines data from diverse tissues and conditions, as well as single-cell assays, including sci-RNA-seq, Perturb-seq, scRNA-seq and sci-ATAC-seq.
Data exclusions	No data was excluded from public data sets beyond described pre-processing steps (see Methods and reproducibility code).
Replication	Reproducibility was examined using different data sets and settings. We also provide a reproducibility repository which allows recovering reported results. Experiments evaluating accuracy in perturbation response were performed over n=10 different random seeds.
Randomization	This study did not include data collection and thus randomization procedures were not applied. Samples order is not relevant for the computational analysis performed.
Blinding	In the analysis of datasets focused on extracting biological insight blinding was not performed, as the analysis is exploratory, aimed at exposing unknown features in advance. For accuracy in perturbation response samples of held out perturbations were not provided in model training.

Reporting for specific materials, systems and methods

We require information from authors about some types of materials, experimental systems and methods used in many studies. Here, indicate whether each material, system or method listed is relevant to your study. If you are not sure if a list item applies to your research, read the appropriate section before selecting a response.

Materials & experimental systems

n/a	Involved in the study
☒	☐ Antibodies
☒	☐ Eukaryotic cell lines
☒	☐ Palaeontology and archaeology
☒	☐ Animals and other organisms
☒	☐ Clinical data
☒	☐ Dual use research of concern

Methods

n/a	Involved in the study
☒	☐ ChIP-seq
☒	☐ Flow cytometry
☒	☐ MRI-based neuroimaging
